# ColoCap: determining the diagnostic accuracy of colon capsule endoscopy compared with standard colonoscopy in patients at risk of colorectal disease – a study protocol

**DOI:** 10.1136/bmjopen-2025-104661

**Published:** 2025-09-30

**Authors:** Hussain Ibrahim, Monica Haritakis, Lisa Ballantine, Kirsty McCormack, Seonaidh Cotton, Jemma Hudson, Karl Atkin, Stephen Rogers, Lisette Sheena Nixon, Angel Verghese, Hayden Holmes, Shaun Treweek, Graeme MacLennan, Sunil Dolwani, Georgina Gardner, Chris Hurt, Angus Watson, James Turvill

**Affiliations:** 1 University of Aberdeen, Aberdeen, UK; 2 York and Scarborough Teaching Hospitals NHS Foundation Trust, York, UK; 3 CHaRT - Health Services Research Unit, University of Aberdeen, Aberdeen, UK; 4 Health Services Research Unit, University of Aberdeen, Aberdeen, UK; 5 Health Services Research Unit, University of Aberdeen College of Life Sciences and Medicine, Aberdeen, UK; 6 Department of Health Sciences, University of York, York, UK; 7 PPI Representative, York, UK; 8 Centre for Trials Research, Cardiff University, Cardiff, UK; 9 York Health Economics Consortium Ltd, Heslington, UK; 10 Department of Gastroenterology, Cardiff and Vale UHB, Cardiff, UK; 11 Cardiff University, Cardiff, UK; 12 University of Southampton, Southampton, UK

**Keywords:** Patients, Endoscopy, Clinical Trial, Colorectal surgery, GASTROENTEROLOGY

## Abstract

**Background:**

Lower gastrointestinal symptoms attributed to colorectal disease are common. Early diagnosis of serious colorectal disease such as colorectal cancer (CRC), precancerous growths (polyps) and inflammation is important to ensure the best possible outcomes for a patient. The current ‘gold standard’ diagnostic test is colonoscopy. Colonoscopy is an invasive procedure. Some people struggle to cope with it and require intravenous sedation and/or analgesia. It is also resource-intensive, needing to be performed in specialist endoscopy units by a trained team. Across the UK, the demand for colonoscopy is outstripping capacity and the diagnosis of colorectal disease is being delayed. A colon capsule endoscope (CCE) is an alternative colorectal diagnostic. It is a ‘camera in a pill’ that can be swallowed and which passes through the gastrointestinal tract, obtaining visual images on the colon. There is now established experience of CCE in the UK. CCE might provide a less invasive method to diagnose colorectal disease if found to be accurate and effective and provide a means by which to increase the National Health Service (NHS) diagnostic capacity.

**Aims and objectives:**

The aim of this study is to determine the diagnostic accuracy of CCE when compared with colonoscopy in representative and clinically meaningful cohorts of patients. An evaluation of the experiences of CCE for the patient and clinical team and an assessment of cost effectiveness will be undertaken.

**Methods:**

We will undertake three research workstreams (WS). In WS1, we shall perform a paired (back-to-back) study. Each participant will swallow the CCE and then later on the same day they will have a colonoscopy. The study has been designed in collaboration with our Patient Advisory Group and as closely mirrors standard care as is possible. 973 participants will be recruited from three representative clinical contexts; suspected CRC, suspected inflammatory bowel disease and postpolypectomy surveillance. Up to 30 sites across the UK will be involved to maximise inclusivity. Measures of diagnostic accuracy will be reported along with CCE completion rates, number of colonoscopy procedures potentially prevented and adverse events, such as capsule retention. A nested substudy of intraobserver and interobserver agreement will be performed. WS2 will develop models of cost-effectiveness and WS3 will evaluate the patient and clinician experience, with reference to acceptability and choice.

**Anticipated impact:**

The study findings will provide the evidence base to inform future colorectal diagnostic services.

**Ethics and dissemination:**

The study has approval from the North East—Tyne and Wear South research ethics committee (REC reference 24/NE/0178, IRAS 331349). The findings will be disseminated to the NHS, National Institute for Health and Care Excellence, other clinical stakeholders and participants, patients and the public.

**Trial registration number:**

ISRCTN16126290.

**STRENGTHS AND LIMITATIONS OF THIS STUDY**

- This is the largest study of its kind looking at the diagnostic accuracy of colon capsule endoscopy (CCE) in a paired direct comparison with the gold standard, colonoscopy.
- The UK-wide, multicentre study design will allow us to recruit a wide range of participants, increasing the generalisability of its findings.
- ColoCap is designed to look at the diagnostic accuracy of CCE for colorectal lesions in general but is not powered to look at specific colorectal disease, like cancer.

## Background and aims

 Early diagnosis of serious colorectal disease such as colorectal cancer (CRC), precancerous growths (polyps) and inflammation is important to ensure the best possible outcomes for a patient.^1^

Colonoscopy is the mainstay of colorectal investigation in the UK with thousands of tests performed every week.^2^ Direct visualisation of the mucosa, as well as the ability to perform biopsies and deliver therapeutics such as polypectomy, makes it the ‘gold standard’ diagnostic test.^3 4^

Colonoscopy requires thorough bowel preparation and is an invasive, frequently painful test, which carries a small risk of bleeding and perforation.^5^ Many patients require intravenous analgesia and/or sedation to be able to tolerate it. Colonoscopy is a resource-intensive procedure requiring formal training and is generally performed in centralised secondary care endoscopy units. Sometimes a colonoscopy cannot be adequately completed and, while quality and safety parameters in colonoscopy have been developed, significant disease can still be missed.^6^

In the UK, the major demand for colonoscopy is as a diagnostic tool.^7^ Since the COVID-19 pandemic, the demand for diagnostic colonoscopy has continued to increase and exceeds the capacity available to meet the targets for timeliness in CRC and inflammatory bowel disease diagnosis and in premalignant polyp surveillance.^8^,^12^ This places patients at risk.^8 10 11^

Colon capsule endoscopy (CCE) is an alternative colorectal diagnostic investigation that might provide additional capacity as a filter test.^13 14^ It is a ‘camera in a pill’ that can be swallowed, and which passes through the gastrointestinal tract, obtaining visual images of the colorectum. There is now an established experience of using CCEs in the UK.^15 16^ As with colonoscopy, the colon needs to be fully clean before CCE is performed.^17^,^19^ Clear fluids and purging laxatives need to be taken before the procedure. This can be difficult for some to tolerate, but the CCE itself rarely causes side effects, such as pain or vomiting.^15 20^

CCE might, therefore, provide a less invasive method to diagnose colorectal disease if found to be accurate and effective, and it may also provide a means by which to increase National Health Service (NHS) diagnostic capacity.

ColoCap aims to determine the diagnostic accuracy of CCE for the detection of visible mucosal colorectal lesions compared with colonoscopy,^21^,^23^ to assess intraobserver and interobserver variability in CCE reading, to assess the cost-effectiveness of CCE in clinical settings, and to evaluate the experience of patients and clinicians using CCE.

## Methods

We will undertake three research workstreams (WS).

### Diagnostic accuracy study (WS1)

This will be a multicentre, paired (‘back-to-back’) study with each participant consenting to undergo a CCE, followed on the same day by the colonoscopy that is part of their standard care.

#### Population

Patients will be recruited from up to thirty units across England, Wales and Scotland. Hospitals that have an existing CCE service or are in the process of setting up a CCE service and have the capacity and capability to take part in this study will be invited to submit expressions of interest.^16 24^ We shall purposefully recruit from sites across England, Scotland and Wales that serve ethnically diverse and socioeconomically disadvantaged populations or those with geographic accessibility challenges. Eligibility criteria are outlined in box 1.

Box 1 **Eligibility criteria**

**Inclusion criteria**

- Non-screening patients with suspected colorectal cancer (CRC) who have had a faecal immunochemical test (FIT) within 3 months of referral,^43^ where a new IBD colitis is suspected^44^ or patients having a 3-yearly postpolypectomy surveillance colonoscopy.^45^
- Patients who feel they can tolerate a same day colon capsule endoscope (CCE) and colonoscopy investigation or would be willing to have the colonoscopy on an alternative day within 2 weeks of the CCE procedure.
- Patients who feel able to swallow the CCE.
- Patients able and willing to give informed consent to participate.

**Exclusion criteria**

- Patients <18 years.
- Patients who are unable to safely swallow the CCE.*
- Patients who are unable to safely and fully comply with the bowel preparation.*
- Patients clinically at risk of stricturing bowel disease, such as Crohn’s disease.
- Patients who have ever received abdominal or pelvic external beam radiotherapy.
- Patients with a history of bowel obstruction.
- Patients who have had a (partial) colectomy.
- Patients who are currently pregnant or breastfeeding.
- Symptomatic patients with suspected CRC who have not had an FIT within 3 months of referral.
- Patients with a permanent pacemaker or other implanted electromedical device.
- Patients who will not be able to safely tolerate the study.*
- Patients in whom the bowel preparation for CCE will likely be inadequate.†

*These exclusion criteria will require some clinical judgement in line with the existing approach to CCE and colonoscopy in clinical practice. Judgement of ability to tolerate the study requires an assessment of frailty per se, rather than a specific comorbidity. However, it is likely to include patients with conditions such as cirrhosis, diabetes, stroke, peripheral vascular disease, heart or renal disease or cognitive impairment.^46^

They are also likely to exclude those with allergies to the medication used in the preparation, those who will not be able to comply with study procedures, and those with structural or functional intestinal pathologies such as volvulus, severe adhesions, enteric fistula or megacolon, for example.

†This exclusion criteria will also require some clinical judgement in line with the existing approach to CCE and colonoscopy in clinical practice. It will include patients with slow gastrointestinal motility, such as idiopathic slow transit constipation, those currently using opioid or tricyclic antidepressant medication, a history of prior poor bowel preparation and/or those who require regular laxatives in their daily round.

#### Interventions

##### Bowel preparation

The current bowel preparation regimen used in the UK for CCE evaluations will be followed. This is similar to standard bowel preparation for colonoscopy.

A low residue diet will be taken for up to 5 days and then on the day before the procedure, the participant will be asked not to eat any food. Clear liquids that day will be acceptable and the participant will be encouraged to take approximately eight glasses (240 mL each) of clear liquids. These will help the participant to stay hydrated and start cleaning the colon. At 17:00 on the day before the procedure, the participant will need to drink 1 L of polyethylene glycol (PEG) 3350 plus ascorbate (Moviprep). For sites unable to access Moviprep from their pharmacies, Plenvu could be used as an alternative PEG-based laxative.^25^

The participant will then continue on a clear liquid diet and at 20:00, repeat the steps above and take a second litre of PEG solution. The participant will once again continue with plenty of clear liquids over the evening until going to bed.

On the morning of the day of the procedure, the participant will take 1 or 2 mg prucalopride (a prokinetic),^17 26^ allergies excepted. The 2 mg dose will be offered to older participants or those in whom reduced gut motility is clinically suspected. The CCE can then proceed. After this, the participant should not eat or drink anything until advised by the clinical team. Bowel cleansing will be assessed using Colon Capsule CLEansing Assessment and Report (CC-CLEAR) and Boston bowel preparation scores, respectively, in the CCE and colonoscopy reports.^19 27^

##### Colon capsule endoscope

Currently, the only CCE service widely available in the UK is provided by the PillCam COLON capsule, supplied by Medtronic. But other capsules may enter clinical practice in future, such as the OMOM CC (Jinshan company) and the PC-I manufactured by ANKON Technologies. All CE (conformité européenne)/UKCA (UK Conformity Assessed) marked CCE services with the technical ability for video upload onto accessible reading platform for review would be eligible for evaluation in this study.

On the day of the procedure, safety checks will be conducted and the participant’s preparedness, fitness and consent will be confirmed by the health professional administering the CCE.

A recorder belt containing radio sensors will be fitted. The recorder sits around the waist in a pouch with a strap around the shoulder holding it in place.

45 min after prucalopride, the CCE pill will be swallowed. Once swallowed, the clinical team will check the capsule progress via the recorder to ensure that the equipment is functioning, and that the recorder is wirelessly capturing the images obtained by the capsule. This will take about 30 min and thereafter the participant will be free to return home, should they wish. If the participant returns home, they will be given the ‘boosters’ (additional laxatives to help move the capsule through the bowel) and a bisacodyl suppository to take with them. The belt and recorder will need to be worn until the colon capsule has passed or until they have to attend for their prearranged colonoscopy and the participant will need to be able to follow the procedural instructions to take additional laxatives that act as ‘boosters’ to help move the capsule through the bowel (see below).

The CCE recorder generates ‘alerts’ to prompt the patient to take the ‘booster’ medicines. Since these need to be timed, the participant will have to be able to understand their purpose and comply with the accompanying instructions. They are generally well tolerated but can cause discomfort and diarrhoea. The ‘alerts’ sent by the CCE reader may vary depending on the supplier, but generally they will follow the format detailed below.

Alert 1: once the capsule has entered the small bowel, the participant will receive this alert and should take a booster. This booster will contain 30 mL of the sodium phosphate solution and 50 mL of gastrografin. Since the booster helps propel the capsule into and through the colon, ready and urgent access to a toilet is a necessity. The participant will continue to take about one litre of water over the next hour, remain active and wait for the next alert.^28^

The next alert will be either ‘end of procedure’ or ‘alert 2’. End of procedure indicates to the patient that the CCE has been excreted.

Alert 2: Three hours after ‘alert 1’ the participant may receive this alert and should take the second smaller booster. This is 15 mL of sodium phosphate mixed with water and 50 mL of gastrografin. The participant should continue drinking about 0.5 L of water over the next hour and wait for the next alert.

The next alert will either be ‘end of procedure’ or ‘alert 3’.

Alert 3: 2 hours after ‘alert 2’, the participant will receive this alert if the capsule has not been excreted. Here, the 10 mg bisacodyl suppository should be inserted into the rectum to allow the capsule to be excreted.

End of procedure: This alert will prompt the participant to contact the clinical team and to attend the endoscopy unit for a colonoscopy. At this stage, the participant should not eat or drink. The participant can remove the belt and be recorded at this stage.

The capsule usually passes in 4–6 hours and based on current experience, we anticipate 80%–85% of capsules to have passed within 8 hours. The capsule is disposable and can be flushed away safely down the toilet when it passes. Adequate CCE completion is defined as a test that visualises the entire length of the colon and the anal cushions, or is excreted within the capsule’s battery life.

##### Colonoscopy

Later in the afternoon, the participant will attend the endoscopy unit where a colonoscopy will be performed. If the capsule has not been excreted by 16:00, the participant should still attend the endoscopy unit for a colonoscopy. The belt and recorder will be removed at the endoscopy unit. It is safe to perform the colonoscopy with the CCE still within the bowel. Colonoscopy is considered complete when the entire length of the colon is intubated as confirmed by features visualised in the caecum (appendix orifice, tri-radiate fold and the ileo-caecal valve).

Recovery and patient follow-up will follow standard clinical practice.

All makes of colonoscope will be allowed in this study. Key performance indicators of colonoscopy quality and safety exist as part of accreditation for colonoscopy in the UK and these will be recorded for all colonoscopists who take part in this study. In newer colonoscopes, additional optical facilities may be available, such as ‘Narrow Band Imaging’, ‘Texture and Colour Enhancement Imaging’ and a ‘computer-aided detection application’ that uses artificial intelligence (AI) to increase polyp detection rate (ENDO-AID CADe). The use of these facilities will be recorded if used during the colonoscopy.

A provisional colonoscopy report is ordinarily provided to the patient immediately after the procedure. This study compares diagnostic accuracy by optical means, although colonoscopic findings may be supported by subsequent histology. The histology from any biopsies or polypectomies will be reconciled when available. The CCE reader will be masked from the colonoscopy result. If the CCE reader becomes unmasked to the colonoscopy prior or during the reading, this will be considered a protocol breach.

It is possible that the participant feels unable to proceed to a same-day colonoscopy. That being the case, efforts will be made to offer a next day or deferred colonoscopy (within 2 weeks of the CCE). Nonetheless, some participants may not feel able to proceed to that colonoscopy. Based on a review of the literature, we estimate that this may occur in up to 10% of cases.^23 29^ Our aim is that by providing potential participants with sufficient information and support, this number can be kept to a minimum.

After the CCE is complete, the research team will supervise the downloading of the data to a secure computer and co-ordinate the clinical analysis of the images into a video for review. Trained and approved CCE readers will record all colorectal findings. Mucosal lesions will be defined by their size, site, extent and characteristics, as appropriate. CCE results will be available within 7 working days.^30 31^

Participants will be informed of the CCE findings in line with standard clinical care at each site. The findings will be explained, discussed and addressed as outlined above and unblinded to the colonoscopist at this stage, as necessary.

Key patient, investigative and clinical outcome data (including adverse events) will be uploaded onto an electronic case report form (eCRF). This will include quality standards for both tests.

### Outcomes

The primary outcome is the diagnostic accuracy of CCE compared with colonoscopy. Diagnostic accuracy will be measured by comparing the per-patient detection of the combined endpoint of visible mucosal colorectal lesions (CRC, polyps and colitis) by CCE and colonoscopy in participants who have had a complete and adequately prepared CCE and colonoscopy.^32 33^

Secondary outcomes will be the diagnostic accuracy for specific lesion types including all polyps and by size (<6 mm, 6–9 mm and >9 mm) and per-lesion matching. For CCE, completion rates and times, bowel preparation adequacy rates, retention rates and adverse events will be recorded, while for colonoscopy it will be standard performance measures and adverse events. We will also assess CCE performance characteristics compared with colonoscopy based on patient demographics, faecal immunochemical test (FIT) and other disaggregated groups. A supplementary ‘intention to investigate’ comparative analysis of CCE versus colonoscopy will be undertaken.

### Sample size calculation and statistical analysis

The sample size calculation is primarily driven by the requirements to test the sensitivity of CCE compared with colonoscopy. We followed the approach recommended by Chu and Cole because the expected sensitivity is high.^34^ The statistical test that is used is a test of a single-sample proportion using an exact binomial test:

H0: p=p0 vs Ha: p>p0

Where p0 is the null sensitivity proportion we wish to rule out (set to 0.90). Following Chu and Cole, for 90% power, a one-sided 5% alpha, and an expected sensitivity of 0.95, 263 disease positive cases are required. 263 cases provide 93% power, but due to the sawtooth nature (ie, non-monotonic) of the power function for an exact test of a single proportion, Chu and Cole recommend taking the lowest sample size N such that the required power (here 90%) is guaranteed for sample sizes larger than N.

We expect a colorectal disease prevalence of 40% in the cohort recruited.^15 16^ Using the formula N_controls=N_cases [(1-Prev)/Prev] where N_cases is the required number of cases and Prev is the expected prevalence, we derive the number of disease negative controls as 394 and a total sample size of 263+394=657. 394 disease negative controls give above 90% power to rule out 75% specificity given an expected specificity of 85%.

We anticipate 10% of participants who agree to take part will subsequently decline one or other of CCE and colonoscopy, as seen in comparable European studies.^21^,^2329^ Also, we anticipate that in 25% cases where both CCE and colonoscopy are achieved, results of one or other of the tests will be incomplete or inadequately prepared. Therefore, to get 657 paired test results, we expect that we will need to consent 973 participants.

There will be one final statistical analysis when recruitment is complete, and the study database is cleaned, checked and locked. Only participants with both a complete and adequately prepared CCE and colonoscopy will be included in the primary analysis to be used to determine accuracy statistics.

The intervention is a CCE and the reference test is colonoscopy. A test positive result will be the detection of mucosal lesions (CRC, polyps or colitis), at the patient level, by CCE; the colorectal disease positive status will be the detection of any mucosal lesion at the patient level, by colonoscopy. The index test accuracy (its sensitivity and specificity) will be assessed in the first instance based on a one-sided comparison between the results of the intervention and those of the reference standard. The statistical test is one-sided because this approach assumes that any discrepancy is an error in the index test. The proportion of participants with inconclusive results, due to an incomplete study and/or inadequate bowel preparation, will be reported and their impact on estimates of test accuracy will be assessed by including them as either test positives or test negatives in the sensitivity analyses.

We know that colonoscopy diagnosis is not 100% accurate and ignoring this could underestimate the value of CCE.^23^ To account for this, we will compare CCE findings with the final diagnosis and also use correction methods from Umemneku Chikere *et al*’s review of methodology to deal with diagnostic tests in the absence of a gold standard.^35^ Final diagnosis of identified mucosal lesions will be informed by relevant histology and a possible second colonoscopy. We will also report specificity, sensitivity, positive and negative predictive values, and likelihood ratios along with their two-sided 95% CIs from this analysis.

Secondary outcomes results will also be analysed and reported separately by specific lesion types and size, on a per-lesion matching basis, on the three patient groups (including FIT) and on other disaggregated groups based on CCE performance characteristics such as age and sex. Other outcomes to be reported are completion and bowel preparation adequacy rates and adverse events. All outcomes will be reported by indication.

### Planned recruitment rate

The recruitment projection is based on an estimate of 30 active centres contributing 2 participants per month over 24 months (4 eligible patients per month; 50% willing to be recruited).

### Monitoring

#### Internal pilot

An internal pilot stage has been included in the study. This 9-month pilot will commence in January 2025 and run to the end of September 2025.

#### Adverse events

All adverse events will be reported to the sponsor as soon as details are available. The chief investigator (CI) will provide clinical oversight of the safety of patients participating in the trial, including an ongoing review of the risk/benefit. Using medical judgement, the adverse events will be assigned seriousness, causality and whether the event/reaction was expected using a predefined set of conditions detailed in the Reference Safety Information approved for the trial. The data monitoring committee will periodically review overall safety data to determine patterns and trends of events, or to identify safety issues, which would not be apparent on an individual case basis. Participants will be followed up until the adverse reaction has resolved or a final outcome has been reached.

Adverse events are likely to be rare. The capsule cannot be swallowed or is vomited up by the patient on 0.2% of occasions. Once the capsule has been swallowed, adverse events, such as pain, occur on <1% of occasions.^15 20 36^ Retention of a capsule is even rarer (3:1000) but would necessitate additional assessment and likely X-rays, such as a plain abdominal X-ray followed by a CT of the abdomen and pelvis, should the retention be confirmed. Retention is defined by the failure of the CCE to be excreted 14 days after swallowing it and is invariably caused by bowel pathology causing an unanticipated stricture. Onward management would be on clinical grounds but is usually dependent on the identification of that previously undiagnosed stricture usually caused by inflammatory bowel disease (IBD) (Crohn’s disease) or CRC. MRI scans cannot be performed on patients until the excretion of the capsule has been formally documented.

The abdominal X-ray will be performed only if there is a clinical suspicion of capsule retention due to obstruction. The X-ray will be performed in accordance with the Ionising Radiation (Medical Exposure) Regulations 2000.

#### Repeat colonoscopy for potentially missed colorectal disease

For some, the CCE may identify a polyp ≥6 mm, or other clinically significant pathology, that is not seen at the subsequent colonoscopy.^37^ This will prompt the consideration for a repeat colonoscopy within 6 weeks, should it be clinically indicated. Here the CCE findings will be unblinded to the colonoscopist and the findings of the repeat procedure will be recorded in the eCRF.

### Substudies and other WS

In addition to the diagnostic accuracy study outlined above in WS1, ColoCap will also include other substudies and WS (further details of which can be found in the online supplemental data) summarised below:

- An intraobserver and interobserver variation study is included,^38 39^ where 140 CCE videos will be assessed by a second reviewer, and an additional 140 will be reassessed by the first reviewer. The reviewers will be blinded to the colonoscopy results and will not know if they are reviewing the video for the first time.
  We will describe the intraobserver and interobserver variation of mucosal lesions, bowel preparation adequacy and completion as well as the improvement in accuracy that can be achieved for the above endpoints by double read of colon capsule videos. The degree of reviewer agreement will dictate the variability with which CCE can reliably prevent the need for colonoscopy.
- WS2, an economic evaluation, will estimate the cost-effectiveness of CCE when compared with colonoscopy. We will develop three health economic models to evaluate the costs and benefits of CCE in symptomatic, surveillance and IBD populations. The intervention is defined as CCE, the tested diagnostic, with follow-up colonoscopy as required based on the pathology identified or the incompleteness/inadequacy of the CCE examination. The comparator is colonoscopy alone, the current standard of care for the diagnosis of CRC, polyps and IBD colitis.
- WS3, a qualitative evaluation of patient and clinician experience, will explore patient preference and acceptability, with a focus on what a CCE diagnostic service should look like from a patient perspective.^40^ This telephone interview will focus on patients experience of CCE, their expectations and their perceived value of the test.^41^ Additionally, interviews will be conducted with 20 clinicians drawn from different parts of the UK who will be selected according to different degrees of enthusiasm for CCE diagnostics. These will explore care professionals’ experience of negotiating the use of CCE, including identifying potential patient barriers/facilitators and second, discussing their response to the findings exploring patient experience.

## Ethical and regulatory considerations

### Consent

Consent to enter the ColoCap study will be undertaken separately from the clinical consenting process for CCE and colonoscopy. For the latter, participants will be consented in line with existing Trust/Board clinical services.

Since there will be significant clinical variability in the initial assessment and scheduling of the colonoscopy and CCE procedures, potential participants can be approached, informed and consented through a range of available methods.

Eligible patients can be approached by a member of their clinical care team either prior to, or at, the patients preassessment appointment. This approach can be either by post, phone, virtually or face-to-face.

Potential participants will be provided with information about the study and will be asked whether they would be happy to be contacted by a member of the research team by telephone to discuss the study further. The research team will determine if the potential participant meets the eligibility criteria.

The consenting process will carefully explore with potential participants the nature and procedure of the study outlines above, as well as the additional risks and benefits of including a CCE, and the unlikely possibility of needing a subsequent colonoscopy should significant pathology be missed by the initial colonoscopy.

Patients who are happy to take part in the study will be invited to consent. At a minimum, the research team must receive verbal consent from the patient to join the ColoCap study, to enable the clinical team to schedule the CCE procedure. This verbal consent must be documented in the patients’ medical notes. Written consent can subsequently be obtained by post, email or on the day of the procedure.

### Research ethics committee review and reports

The study has approval from the North East—Tyne and Wear South research ethics committee (REC reference 24/NE/0178, IRAS 331349). Substantial amendments that require review by the REC will not be implemented until the REC grants a favourable opinion for the study. The chief investigator will notify the REC of the end of the study. If the study is ended prematurely, the chief investigator will notify the REC, including the reasons for the premature termination.

### Peer review 

As part of the funding process, the National Institute for Health and Care Research (NIHR) sought external reviews on the study proposal, which resulted in the study being funded.

The study was peer-reviewed by the research and development (R&D) Group at York and Scarborough Teaching Hospitals NHS Foundation Trust and the Study Adoption Group at Cardiff University.

### Public and patient involvement

This study has been shaped by extensive patient and public involvement. One of the coapplicants on the funding application is a member of York and Scarborough Teaching Hospitals Public Contributor Group. They participated in applicants’ meetings for stage 1 and 2 development, reviewing and commenting from a patient perspective. A Patient Advisory Group (PAG) was established from the stage 1 focus group. Individuals who have had, or been carers of those with, bowel disease or who have had bowel investigations, along with patient representatives on the NHS England CCE Expert Advisory, joined the PAG. We intend to recruit new members from users who have undergone CCE or colonoscopy across the UK. The composition of the group will then better reflect the range of sociodemographic characteristics of people eligible for CCE and colonoscopy.

The PAG will help develop all patient-facing materials, including coproducing the patient interview topic guides, patient information sheets and the patient questionnaires in WS3. The PAG will be involved in identifying patient-centred themes from interviews in WS3.

The PAG will also provide a patient perspective on the write-up of papers, help produce lay summaries and assist in presenting the study findings results at conferences, support groups, charity events and online. They will be integral to the dissemination and implementation phase of the programme. PAG colleagues will receive payment for their time in line with NIHR INVOLVE recommendations.

### Regulatory compliance

The study will not commence until a HRA approval and Favourable REC opinion has been received. Before any site can enrol patients into the study, the CI/principal investigator or designee will ensure that appropriate approvals from participating organisations are in place.

### Data protection and patient confidentiality

The source data for this study will be the patient medical records (eg, letter, clinic notes, laboratory results), capsule software and patient and clinician interview audio and transcripts.

For each participant, the research team will complete eCRFs. These data will be found in the participants’ medical records. REDCap will be the eCRF used for this study and will be managed and maintained by York and Scarborough Teaching Hospitals NHS Foundation Trust. Access to the eCRF will be restricted. At recruiting centres, only authorised personnel will be able to see or make entries or amendments to that site’s patients’ data.

#### Access to data

Direct access will be granted to authorised representatives from the sponsor, host institution and the regulatory authorities to permit trial-related monitoring, audits and inspections in line with participant consent.

#### Archiving

Once the study has ended, sites will be asked to complete an end of study checklist. Once this has been returned, the sponsor will confirm that the study documents can be archived. Study documents will be archived by the participating sites.

Study documents will be archived for 5 years from the end of study as per the sponsors standard operating procedures. After the agreed retention time, the sponsor will authorise the destruction of study documents.

All investigators and trial site staff will comply with the requirements of the Data Protection Act 2018 and the general data protection regulation with regard to the collection, storage, processing and disclosure of personal information and will uphold the Act’s core principles.

#### Dissemination policy

This study has been designed, from the outset, to produce useful, timely and relevant research findings that will allow the rapid implementation of a diagnostic CCE service throughout the UK in support of colorectal disease diagnostics. We have involved the commissioners in all three participating nations during the design of ColoCap and we will communicate with them throughout the course of the study. Through these partnerships, we anticipate providing evidence for actionable findings of immediate utility to decision-makers and service users. The main outputs from our research will be presented in peer-reviewed international journals and disseminated proactively to NHS England, Scotland and Wales as well as to Health and Social Care in Northern Ireland. Working with our PAG and Integrated Care Boards, we will organise dissemination events aimed at patients and the public.

#### Study registration

ColoCap was registered on ISRCTN on 23 October 2024. Registration number ISRCTN16126290.^42^

## Supplementary material

10.1136/bmjopen-2025-104661 online supplemental file 1
