# Supplementary material for: ColoCap: determining the diagnostic accuracy of colon capsule endoscopy compared with standard colonoscopy in patients at risk of colorectal disease – a study protocol
Source: BMJ Open. 2025 Sep 30;15(9):e104661. doi: 10.1136/bmjopen-2025-104661 (PMC12506084; doi:10.1136/bmjopen-2025-104661)
Supplement: online supplemental file 1 [file bmjopen-15-9-s001.pdf]

ColoCap: determining the diagnostic accuracy of colon capsule endoscopy compared to standard colonoscopy in patients at risk of colorectal disease

ColoCap

Professor James Turvill- Co- Chief Investigator

Professor Angus Watson- Co- Chief Investigator

This protocol has regard for the HRA guidance and order of content.

RESEARCH REFERENCE NUMBERS

TRIAL REGISTRY NUMBER AND DATE: ISRCTN16126290 registered on 23/10/2024

PROTOCOL VERSION NUMBER AND DATE: 2.0 (03/02/2025)

OTHER RESEARCH REFERENCE NUMBERS

SPONSOR: York and Scarborough Teaching Hospitals NHS Foundation Trust

FULL/LONG TITLE OF THE TRIAL

ColoCap: determining the diagnostic accuracy of colon capsule endoscopy compared to standard colonoscopy in patients at risk of colorectal disease.

SHORT TRIAL TITLE / ACRONYM

ColoCap

PROTOCOL VERSION NUMBER AND DATE

2.0

3rd February 2025

RESEARCH REFERENCE NUMBERS

**IRAS Number:**	**331349**
**ISRCTN Number / Clinical trials.gov Number:**	ISRCTN16126290
**FUNDERS Number:**	NIHR158034

SIGNATURE PAGE

The undersigned confirm that the following protocol has been agreed and accepted and that the Chief Investigators agree to conduct the trial in compliance with the approved protocol and will adhere to the principles outlined in the UK Policy Framework for Health and Social Care Research, Good Clinical Practice (GCP) guidelines, the Sponsor's (and any other relevant) Standard Operating Procedures (SOPs), and other regulatory requirements as amended.

I agree to ensure that the confidential information contained in this document will not be used for any other purpose other than the evaluation or conduct of the clinical investigation without the prior written consent of the Sponsor.

I also confirm that I will make the findings of the trial publicly available through publication or other dissemination tools without any unnecessary delay and that an honest accurate and transparent account of the trial will be given; and that any discrepancies and serious breaches of GCP from the trial as planned in this protocol will be explained.

For and on behalf of the Trial Sponsor:

Signature: .... Date: ....../....../......

Name: [redacted]

Position: RESEARCH ADVISOR

Co-Chief Investigator:

Signature: .... Date: ....../....../......

Name: PROFESSOR JAMES TURVILL

Co-Chief Investigator:

Signature: .... Date: ....../....../......

Name: PROFESSOR ANGUS WATSON

Co-Investigator:

Signature: .... Date: ....../....../......

Name: PROFESSOR SUNIL DOLWANI

Statistician:

Signature:
....

Name: (please print):

....

Position:
....

Signature:
....

Name: (please print):

....

Position:
....

PI SIGNATURE

Principal Investigator (PI) responsibilities

The PI's legal responsibilities are clearly defined in the UK Policy Framework for Health and Social Care Research & Clinical Trials Regulations.

As PI I take responsibility for the conduct of the research at the site and must ensure that the research is conducted according to the approved protocol and in compliance with any applicable regulatory standards and guidance. This includes:

- Attending the site initiation visit
- Ensuring new members of the trial team are appropriately trained in the protocol and its procedures
- Ensuring that the ISF is accurately maintained
- Disseminating important safety or trial related information to all stakeholders within their site
- Ensuring that safety reporting is completed within the timelines
- Ensuring data is submitted to the eCRF in a timely manner

Principal Investigator:

Signature: .... Date: ....../....../......

Name:

KEY TRIAL CONTACTS

	[redacted]<br>[redacted]
	[redacted]
Co- Investigator	[redacted]
	[redacted]
Sponsor	[redacted]
Funder(s)	[redacted]
Clinical Trials Unit	[redacted]

[redacted]

	[redacted]
[redacted]	[redacted]
[redacted]	[redacted]
[redacted]	[redacted]

[redacted]

Amendment History

Amendment No.	Protocol version no.	Date issued	Author(s) of changes	Details of changes made
Amendment 5	2.0	03/02/2025	Monica Haritakis	Addition of funder acknowledgement.<br>Updating the SWAT to a choice of vouchers and a travel SWAT.<br>Other minor updates.

i. LIST of CONTENTS

GENERAL INFORMATION	Page No.
TITLE PAGE	3
RESEARCH REFERENCE NUMBERS	4
SIGNATURE PAGE	5
KEY TRIAL CONTACTS	8
i. LIST of CONTENTS	11
ii. LIST OF ABBREVIATIONS	12
iii. TRIAL SUMMARY	14
iv. FUNDING	17
v. ROLE OF SPONSOR AND FUNDER	17
vi. ROLES & RESPONSIBILITIES OF TRIAL MANAGEMENT COMMITTEES, GROUPS AND INDIVIDUALS	17
viii. KEYWORDS	17
ix. TRIAL SCHEMA	18
x. SCHEDULE OF EVENTS	19
**SECTION**	
1. BACKGROUND	20
2. RATIONALE	20
3. OBJECTIVES AND OUTCOME MEASURES/ENDPOINTS	21
4. TRIAL DESIGN	24
5. TRIAL SETTING	25
6. PARTICIPANT ELIGIBILITY CRITERIA	25
7. TRIAL PROCEDURES: WS1	26
8. TRIAL INTERVENTION: WS1	29
9. SAFETY REPORTING	34
10. SUB STUDIES	38
11. STATISTICS AND DATA ANALYSIS	43
12. DATA MANAGEMENT	46
13. MONITORING, AUDIT & INSPECTION	46
14. ETHICAL AND REGULATORY CONSIDERATIONS	46
15. DISSEMINATION POLICY	50
16. REFERENCES	51
17. APPENDICIES	57

ii. LIST OF ABBREVIATIONS

Define all unusual or 'technical' terms related to the trial. Add or delete as appropriate to your trial. Maintain alphabetical order for ease of reference.

AE	Adverse Event
AR	Adverse Reaction
CCE	Colon Capsule Endoscopy
CI	Chief Investigator
CRC	Colorectal Cancer
CRF	Case Report Form
CRO	Contract Research Organisation
CTU	Clinical Trials Unit
DMC	Data Monitoring Committee
EC	European Commission
EU	European Union
FIT	Faecal Immunochemical Test
GCP	Good Clinical Practice
IBD	Inflammatory Bowel Disease
ICF	Informed Consent Form
ISF	Investigator Site File (This forms part of the TMF)
ISRCTN	International Standard Randomised Controlled Trials Number
MS	Member State
NHS R&D	National Health Service Research & Development
PAG	Patient Advisory Group
PI	Principal Investigator
PIC	Participant Identification Centre
PIS	Participant Information Sheet
QA	Quality Assurance
QC	Quality Control
QP	Qualified Person
REC	Research Ethics Committee
SAE	Serious Adverse Event
SDV	Source Data Verification
SOP	Standard Operating Procedure
SSI	Site Specific Information
SWAT	Study Within A Trial

TMF	Trial Master File
TMG	Trial Management Group
TSC	Trial Steering Committee

iii. TRIAL SUMMARY

Trial Title	ColoCap: determining the diagnostic accuracy of colon capsule endoscopy compared to standard colonoscopy in patients at risk of colorectal disease.	
Short title	ColoCap	
Trial Design	A multicentre study comparing Colon Capsule Endoscopy (CCE) to colonoscopy, performed 'back to back', for the detection of visible mucosal colorectal lesions (CRC, polyps and colitis).<br><br>An assessment of the cost-effectiveness of CCE when compared to colonoscopy and an evaluation of the experiences of CCE for the patient and the multi-professional team compared to colonoscopy, will be undertaken.	
Trial Participants	Patient groups: three representative patient groups will be selected for recruitment:<br>• symptomatic patients referred with suspected CRC; this group will be subdivided, based on FIT into a higher risk sub-group (FIT≥10µg Hb/g faeces) and a lower risk sub-group (FIT<10µg Hb/g faeces).<br>• patients where a new IBD colitis (usually ulcerative colitis (UC)) is suspected (note that not all patients with suspected IBD will be recruited because of the risk of bowel stricturing (narrowing) associated with Crohn's disease),<br>• patients awaiting a 3 yearly post-polypectomy surveillance colonoscopy	
Planned Sample Size	973 participants consented.<br><br>(657 participants with a complete and adequate CCE and colonoscopy)	
Intervention duration	1 day	
Follow up duration	Until diagnosis	
Planned Trial Period	Planned study start date: 01/04/2024<br><br>Planned study end date: 30/09/2027<br><br>Recruitment start date: 01/01/2025<br><br>Recruitment end date: 31/12/2026	
	Objectives	Outcome Measures
Primary	Diagnostic accuracy of CCE compared to standard colonoscopy.<br><br>The analysis will be performed on participants	Per-patient detection of the combined endpoint of visible mucosal lesions (CRC, polyps and colitis). The per-patient basis analysis has been selected since it informs

	who have had complete examinations with adequate bowel preparation.	the decision for onward intervention.<br><br>Further analyses will be performed on an 'intention to investigate' basis and post review colonoscopy, when appropriate (see below).<br><br>Secondary outcome measures on the primary objective will be performed for specific lesion types (polyps combined and <6, 6-9, >9mm in size), on a per-lesion matching basis, on patient groups, FIT and other disaggregated groups based on CCE performance characteristics.<br><br>For CCE, completion rates and times, bowel preparation adequacy rates, retention rates and adverse events will be recorded while for colonoscopy it will be standard performance measures and adverse events.
Secondary	An assessment of CCE intra- and inter- reader variability.	A comparative evaluation of outcomes will be conducted. This will be for visible mucosal lesions (CRC, polyps and IBD colitis) and measures of bowel preparation adequacy and completion. The impact of that variability on diagnostic accuracy (primary outcome) will be assessed.<br><br>Subgroup analysis of specific lesions will be performed as

		outlined above. The improvement in accuracy that can be achieved for the above endpoints by blinded complete double read of colon capsule videos will be described.
	To develop health economic models to evaluate the costs and benefits of CCE in relevant patient groups.	For patients undergoing CCE for suspected CRC or post polypectomy surveillance, the key outputs of the models will be the total incremental costs, total incremental QALYs and life-years, cost per colonoscopy avoided, and the excess number of CRC detected.<br><br>For those having a CCE for suspected colitis a further model will capture the economic impact of using CCE within the diagnostic pathway for IBD.
	To evaluate the patient and clinician experience of CCE.	To provide a thematic account, using patient and clinician experience, to help understand the findings derived from the above stated objectives. In presenting our analysis we will further contextualise the experience of our sample by comparing it to the experience of those using colonoscopy, as established by the literature.

iv. FUNDING AND SUPPORT IN KIND

FUNDER(S)<br>(Names and contact details of ALL organisations providing funding and/or support in kind for this trial)
NIHR Health Technology Assessment (HTA) Programme

This research was funded by the NIHR (NIHR158034) using UK international development funding from the UK Government to support global health research. The views expressed in this publication are those of the author(s) and not necessarily those of the NIHR or the UK government.

v. ROLE OF TRIAL SPONSOR AND FUNDER

The Sponsor of this study is York and Scarborough Teaching Hospitals NHS Foundation Trust. The Sponsor assumes overall responsibility for the initiation, management of the study design, conduct, data analysis and interpretation, manuscript writing, and dissemination of results.

The study is funded through an NIHR Health Technology Assessment (HTA) grant.

vi. ROLES AND RESPONSIBILITIES OF TRIAL MANAGEMENT COMMITEES/ GROUPS & INDIVIDUALS

Trial Management Committees

A Trial Management Group (TMG), an independent Trial Steering Committee (TSC), an independent Data Monitoring Committee (DMC) and the Patient Advisory Group (PAG) will be convened.

A TMG will be established to monitor all aspects of the conduct and progress of the trial, ensure that the protocol is adhered to and take appropriate actions to safeguard participants and the quality of the trial itself. The membership and terms of reference of the TMG will be filed in the Trial Master File.

A TSC, with independent members, will be established to oversee the conduct and progress of the trial. The membership and terms of reference of the TSC will be filed in the Trial Master File.

An independent DMC will be established to oversee the safety of subjects in the trial. The membership and terms of reference of the DMC will be filed in the Trial Master File.

A PAG has been established which will be involved in key decisions such as the overarching trial set-up, recruitment, study materials production, data collection, analysis and sharing findings. The membership and terms of reference of the PAG will be filed in the Trial Master File.

**viii. KEY WORDS:**	Colon capsule endoscopy (CCE), colonoscopy, inflammatory bowel disease (IBD), polyps, colorectal cancer (CRC), computed tomography colonography (CTC). Lower gastrointestinal diagnostics

ix. TRIAL SCHEMA

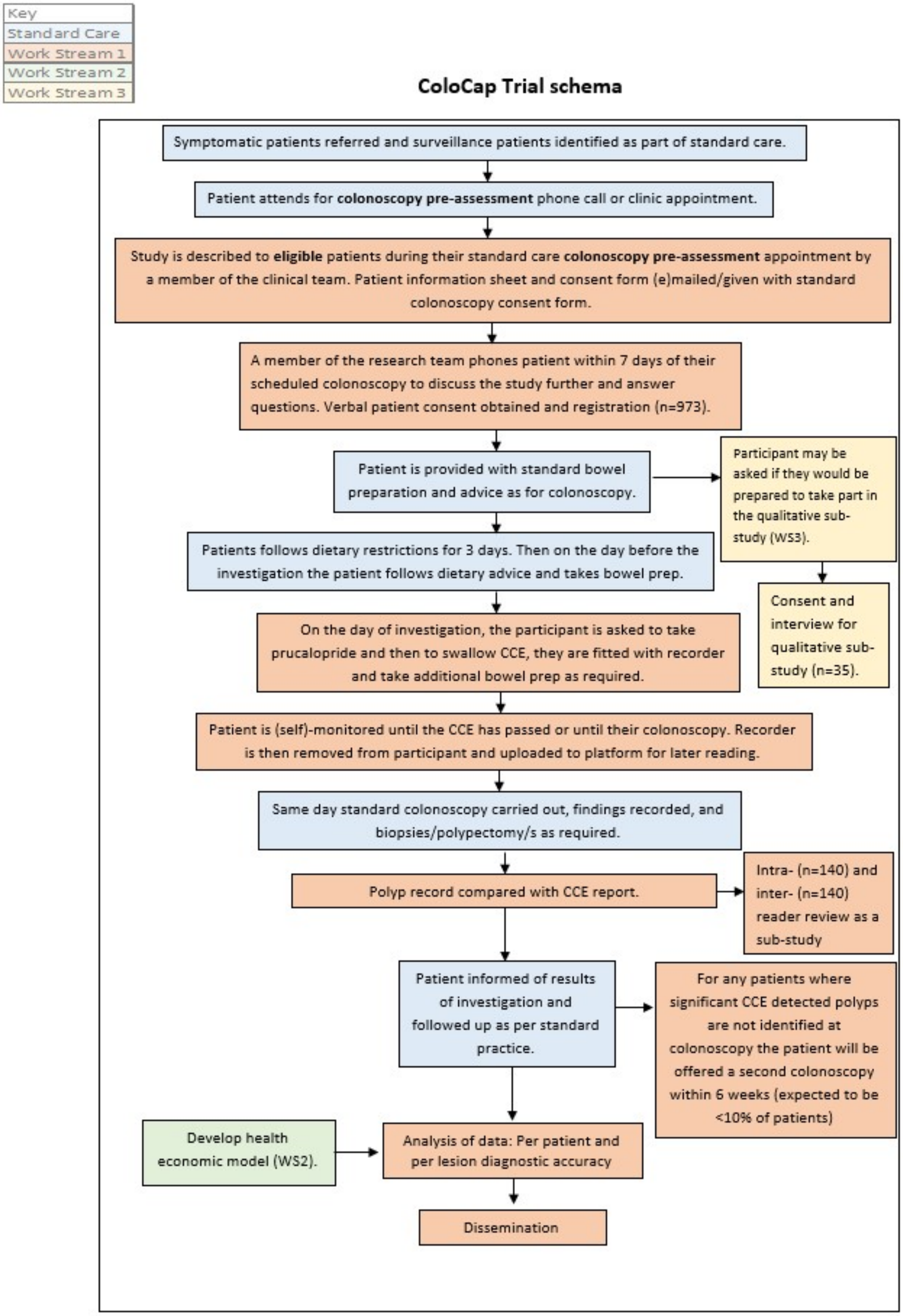

x. SCHEDULE OF EVENTS

Procedures	Day -7 (+/- 60days)	Day -7 (+/- 60 days)	Day -4	Day -3	Day -2	Day -1	Day 0	Day 7 (+/- 7 days)	By Day 42	Final diagnosis (or Day 30)
Eligibility assessment	x	x								
Provision of PIL	x	x								
Informed consent		x					x			
Diet modification			x	x	x	x	x			
Bowel preparation						x (pm)	x			
Colon Capsule Endoscopy							x			
Colon Capsule Report/ results								x		
Standard care Colonoscopy							x			
Standard care Colonoscopy report/ results							x	x		
Repeat colonoscopy: poor bowel prep (Standard Care)*									x	
Repeat colonoscopy: potentially missed colorectal disease *									x	
Demographics (CRF)							x			
Medical and drug history (CRF)							x			
Investigative date (CRF)							x			
Outcome data (CRF)										x
Adverse events (CRF)							x			x

1 BACKGROUND

Early diagnosis of serious colorectal disease such as colorectal cancer (CRC), pre-cancerous growths (polyps) and inflammation is important to ensure the best possible outcomes for a patient. The current 'gold standard' diagnostic test is colonoscopy. Colonoscopy is an invasive and often uncomfortable procedure. Some struggle to cope with it and require intravenous sedation and/or analgesia. It is also resource intensive, needing to be performed in specialist endoscopy units by a trained team. Lower gastrointestinal (GI) symptoms attributed to colorectal disease are common and the diagnostic yield of significant bowel pathology at symptomatic colonoscopy remains relatively modest. Several approaches including introducing a triage or filter test have been considered to improve the effectiveness of colonoscopy by targeting it to those where it is most required and helpful in diagnosis and treatment of bowel disease. Such tests are urgently required since, across the UK, the demand for colonoscopy is outstripping capacity, and the diagnosis of colorectal disease is being delayed.

Colon capsule endoscopy (CCE) is an alternative colorectal diagnostic investigation that might provide additional capacity as a filter test. It is a 'camera in a pill' that can be swallowed, and which passes through the gastrointestinal tract, obtaining visual images of the colorectum. It is a capsule, about 3cm long and 1cm wide, rounded at both ends with a smooth plastic casing. There is now an established experience of using CCEs in the UK. As with colonoscopy the colon needs to be fully clean before taking the CCE. Clear fluids and purging laxatives need to be taken before the procedure. This can be difficult for some to tolerate but the CCE itself rarely causes side effects, such as pain or vomiting. It should not be used in people who might have a narrowing in the bowel that could prevent its passage.

CCE might provide a less invasive method to diagnose colorectal disease if found to be accurate and effective and it may also provide a means by which to increase NHS diagnostic capacity.

2 RATIONALE

Colonoscopy is the mainstay of colorectal investigation and disease diagnosis in the UK with thousands of tests performed every week.[1] Direct visualisation of the mucosa, as well as the ability to perform biopsies, makes it the 'gold standard' diagnostic test. Colonoscopy is used to diagnose diseases such as colorectal cancer (CRC), pre-malignant polyps and inflammatory bowel disease (IBD).[2,3] It is also used for the surveillance of those at risk of developing premalignant polyps and in the monitoring of disease after medical or surgical management.[4] Colonoscopy requires thorough bowel preparation and is an invasive, frequently painful test, which carries a small risk of bleeding and perforation.[7] Many patients require intravenous analgesia and/or sedation to be able to tolerate it. Colonoscopy is a resource-intensive procedure requiring formal training and is generally performed in centralised secondary care endoscopy units. It generates a significant carbon footprint. [8] Sometimes a colonoscopy cannot be adequately completed and, whilst quality and safety parameters in colonoscopy have been developed, significant disease can still be missed.[5] Computed tomographic colonography (CTC) is an alternative colorectal diagnostic that delivers a relatively low radiation dose, although it does not directly visualise the mucosa and biopsies cannot be obtained.[9] It is also an invasive procedure requiring bowel preparation and involving ionising radiation, but it is generally less onerous for the patient to tolerate and lower risk than colonoscopy.[9,10] It too is performed in secondary care. Generally, it is reserved for frailer patients in whom colonoscopy cannot, or should not, be performed.[5,10] Colonoscopy also delivers colorectal therapeutics, such as polypectomy. [3] However, in the UK, the major demand for colonoscopy is as a diagnostic tool.[11] Since the Covid-19 pandemic the demand for diagnostic colonoscopy has continued to increase and exceeds the capacity available to meet the targets for timeliness in CRC and

IBD diagnosis and in premalignant polyp surveillance.[12-18] This places patients at risk.[12,16,17] There is a need to increase diagnostic colorectal capacity and to provide a better tolerated procedure for some. Colon capsule endoscopy (CCE) is a potential alternative.[10,19,20,21]

2.1 Assessment and management of risk

A risk assessment will be carried out by the CIs and reviewed regularly. The risk assessment will be reviewed by the TMG and Sponsor regularly.

3 OBJECTIVES AND OUTCOME MEASURES/ENDPOINTS

The aims of the study are to determine the diagnostic accuracy of CCE for the detection of visible mucosal colorectal lesions (CRC, polyps and colitis) compared to colonoscopy, to assess intra- and inter-observer variability in CCE reading, to assess the cost effectiveness of CCE in clinical settings and to evaluate the experience of patients and clinicians using CCE.[22-24]

3.1 Primary objective

Aim: To compare the diagnostic accuracy of CCE compared to colonoscopy.

This will be a multi-centre, paired (‘back-to-back’) study with each participant consenting to undergoing a CCE, followed by same day colonoscopy as part of standard care (see later for exceptions).

Population: Patient groups: three representative patient groups will be selected:

- Symptomatic patients with suspected CRC; this group will be subdivided, based on FIT into a higher risk sub-group (FIT≥10µg Hb/g faeces) and a lower risk sub-group (FIT<10µg Hb/g faeces).[25]
- Patients where a new IBD colitis (usually ulcerative colitis (UC)) is suspected (note that not all patients with suspected IBD will be recruited because of the risk of bowel stricturing (narrowing) associated with Crohn’s disease).[26, 28]
- Patients awaiting a 3 yearly post-polypectomy surveillance colonoscopy.[4]

3.2 Secondary objectives

- To conduct an intra- and inter-observer sub study. Here we shall describe the intra- and inter-observer variation of CCE reading. This will include variability measures for lesion detection, bowel preparation adequacy and completion. The impact of that variability on diagnostic accuracy (primary outcome) will be evaluated and any improvement in accuracy that can be achieved for the above endpoints by double reading of colon capsule videos will be described. The degree of agreement will dictate the way in which CCE can reliably be used to prevent the need for colonoscopy.[29-31]

- To develop models of cost effectiveness. This workstream (delivered by YHEC) will estimate the cost-effectiveness of CCE when compared to colonoscopy. The decision problem considered will be aligned with the populations that CCE is evaluating. Therefore, three

separate health economic models to evaluate the costs and benefits of CCE will be developed. The evaluation of cost effectiveness will be completed by month 40.[32, 33]

- To perform a qualitative evaluation of the patient and clinician experience of CCE compared to colonoscopy[34]. This will be a synthesised analysis and will explore and explain similarities and differences between the accounts of patients and clinicians, as the basis of understanding accessible and appropriate future care, consistent with best practice.

3.5 Table of endpoints/outcomes

Objectives	Outcome Measures	Timepoint(s) of evaluation of this outcome measure (if applicable)
**Primary Objective**<br>Diagnostic accuracy of CCE compared to colonoscopy.	**Primary outcome:** Per-patient detection of the combined endpoint of visible mucosal colorectal lesions (CRC, polyps and colitis) in participants who have had a complete and adequately prepared CCE and colonoscopy.[35,36] The endpoint will include post review colonoscopy, when appropriate.<br><br>**Secondary outcome:**<br>(i) diagnostic accuracy for specific lesion types including all polyps and by size (<6mm, 6-9mm and >9mm),[37]<br>(ii) per-lesion matching,<br>(iii) for CCE, completion rates and times, bowel preparation adequacy rates, retention rates and adverse events will be recorded while for colonoscopy it will be standard performance	**Timepoints:** Per patient colonoscopy reporting/CCE reading days 0-7.<br><br>Interim analyses: month 12, 18, 24, 30 and 36.<br>Final analysis months 36-40.

	measures and adverse events.[38, 39]<br>(iv) CCE performance characteristics compared to colonoscopy will be assessed based on patient demographics, FIT and other disaggregated groups,<br>(v) a supplementary 'intention to investigate' comparative analysis of CCE versus colonoscopy.	
**Secondary Objectives**<br>Intra- and inter- reader variability.<br><br>Development of health economic models.	**Primary outcomes:**<br>The identification and impact of any variability on diagnostic accuracy of CCE. A structured proforma will be developed to address this.<br><br>To evaluate the costs and benefits of CCE in relevant patient groups.<br>The key outputs of the symptomatic and surveillance CRC models will be:<br>- the total incremental costs,<br>- total incremental QALYs and life-years,<br>- cost per colonoscopy avoided,<br>- the excess number of CRC detected.<br><br>For IBD colitis a third model will be developed to capture the economic impact of using CCE within the diagnostic pathway for IBD	**Timepoints:**<br>Analyses months 28-37.<br><br>Model developments: months 17-37.<br>Analyses: months 38-40.

Evaluation of the patient and clinician experience of CCE.	To provide a thematic account, using patient and clinician experience.	Qualitative analyses months 34-39.
	**Exploratory endpoints/outcomes:**<br>Evaluation of the performance of CCE compared to colonoscopy in each of the three clinical test scenarios (statistically unpowered).<br><br>Exploratory analysis using logistic regression to investigate factors that might influence diagnostic performance of CCE.<br><br>A Study Within A Trial (SWAT) comparing whether offering a choice of £100 vouchers increases recruitment of socioeconomically disadvantaged participants.<br><br>A Study Within A Trial (SWAT) offering door-to-door taxis and its impact on recruitment of socioeconomically disadvantaged participants.	Final analysis months 36-40.<br><br>End of recruitment months 32 - 34

4 TRIAL DESIGN

We will undertake three research workstreams (WS).

In WS1 we shall perform a paired (back-to-back) study. Each participant will swallow the CCE and then, where possible, later in the same day they will have a colonoscopy. The study has been designed in collaboration with our Patient Advisory Group and as closely mirrors standard care as is possible. 973 participants will be recruited from three representative clinical contexts; suspected CRC, suspected inflammatory bowel disease and 3 yearly post-polypectomy surveillance. Up to 30 sites across Great Britain will be involved to help ensure generalisability and inclusivity. Measures of diagnostic accuracy will be reported along with CCE bowel preparation adequacy and completion rates, number of

colonoscopy procedures potentially prevented and adverse events, such as capsule retention. Within WS1, a SWAT and a nested sub-study of intra- and inter-reader agreement will be undertaken.

WS2 will develop models of cost-effectiveness and WS3, patient and clinician experience, with reference to acceptability and choice. WS3 involves a qualitative interview-based evaluation of patient and clinician experience of CCE compared to colonoscopy.

5 TRIAL SETTING: WS1

Recruitment will come from patients at thirty hospital outpatient services and endoscopy units across England, Wales, and Scotland. Hospital Trusts and Health Boards that have an existing CCE service or are in the process of setting up a CCE service and have the capacity and capability to take part in this study will be invited to submit expressions of interest.[40-42] We shall purposefully recruit from sites across the Great Britain that serve ethnically diverse and socioeconomically disadvantaged populations or those with geographic accessibility challenges.

Whilst same day CCE and colonoscopy is anticipated, for pragmatic reasons, options for off- or distant-site CCE followed by same day colonoscopy, or within two weeks, will be explored. The options for delivering CCE beyond the secondary care endoscopy unit setting make it a diagnostic that might favourably influence health seeking behaviour in under-served communities such as socioeconomically disadvantaged, ethnic minorities and rural populations. Currently, general practice, community hospital and home delivery of CCE is taking place in the NHS service evaluations.

5.1 Required documentation from sites

Prior to initiating a participant site the following will be required:

- Completed Expression of Interest form
- Current CV for the PI
- Current GCP certificate for the PI
- Copy of the delegation log
- Signed training log
- Completed OID
- Confirmation of Capacity and capability

6 PARTICIPANT ELIGIBILITY CRITERIA

6.1 Inclusion criteria

- Patients with suspected CRC who have had a FIT within 3 months of referral, where a new IBD colitis is suspected or patients having a 3 yearly post-polypectomy surveillance colonoscopy.
- Patients who feel they can tolerate a same day CCE and colonoscopy investigation or would be willing to have the colonoscopy on an alternative day.
- Patients who feel able to swallow the CCE.
- Patients able and willing to give informed consent to participate.

6.2 Exclusion criteria

- Patients <18 years.
- Patients who are unable to safely swallow the CCE.*
- Patients who are unable to safely and fully comply with the bowel preparation.*
- Patients clinically at risk of stricturing bowel disease, such as Crohn’s disease.
- Patients who have ever received abdominal or pelvic external beam radiotherapy.
- Patients with a history of bowel obstruction.
- Patients who have had a (partial) colectomy.
- Patients who are currently pregnant or breastfeeding.
- Symptomatic patients with suspected CRC who have not had a FIT within 3 months of referral.
- Patients with a permanent pacemaker or other implanted electromedical device.
- Patients who will not be able to safely tolerate the study.*
- Patients in whom the bowel preparation for CCE will likely be inadequate. [x]

* These exclusion criteria will require some clinical judgement in line with the existing approach to CCE and colonoscopy in clinical practice. Judgement of ability to tolerate the study requires an assessment of frailty *per se*, rather than a specific co-morbidity. However, it is likely to include patients with conditions such as cirrhosis, diabetes, stroke, peripheral vascular, heart or renal disease or cognitive impairment.[43-45]

[x]This exclusion criteria will also require some clinical judgement in line with the existing approach to CCE and colonoscopy in clinical practice. It will include patients with slow gastrointestinal motility, such as idiopathic slow transit constipation, those currently using opioid or tricyclic antidepressant medication, a history or prior poor bowel preparation and/or who require regular laxatives in their daily round.

7 TRIAL PROCEDURES

7.1 Recruitment

Patients will be approached through existing clinical pathways to attend for a diagnostic or surveillance colonoscopy.

A convenience series comprising the three study populations will be recruited from multiple sites. It is anticipated that the relative proportion of patients from each patient group will be representative of colonoscopy activity in current clinical practice. We estimate this to be approximately 60% suspected CRC, 30% surveillance and 10% suspected IBD colitis. The TMG will actively monitor patient recruitment to ensure representation into each group noting (i) that the study is powered on the expectation of a colorectal disease prevalence of 40% and (ii) that individual sites may not be in a position to recruit patients from all three referral groups.

Attempts will also be made to reflect a broad mix of recruits in terms of age, gender, ethnicity and socioeconomic status, recognising that these may be influenced by the three referral groups of themselves.

7.2 Participant identification

Individual Trusts/Boards will have different mechanisms for patient identification, assessment and consenting within standard clinical care. Initially, the assessment of suspected CRC patients, those with suspected IBD colitis, and surveillance patients for appropriateness and fitness to tolerate a colonoscopy will be made on clinical grounds.

The clinical care teams at participating sites will initially review patients for study eligibility prior to scheduling their colonoscopy pre-assessment. If the clinical team deems a patient to be potentially eligible, then a member of the clinical team will approach patients.

Recruiting sites will have established processes for communicating with either symptomatic or surveillance patients about their upcoming procedure and this study does not plan to deviate from that but to embed into that process. Allowing sufficient time for completion of the CCE, the subsequent colonoscopy will be scheduled provisionally as a late afternoon procedure. There may be some operational flexibility should the CCE be completed sooner than anticipated, however patients receiving standard care should not have their care disrupted or delayed.

7.3 Consent

Consent to enter the ColoCap study will be undertaken separately from the clinical consenting process for CCE and colonoscopy. For the latter, participants will be consented in line with existing Trust/Board clinical services.

Since there will be significant clinical variability in the initial assessment and scheduling of the colonoscopy and CCE procedures potential participants can be approached, informed and consented through a range of available methods.

Eligible patients can be approached by a member of their clinical care team either:

- Prior to the patients pre assessment appointment
- At the patients pre assessment appointment

This approach can be either by:

- Post
- Phone
- Face to face
- Virtually

Patients being identified and approached by a member of the clinical care team will be provided with information about the study and will ask the patient whether they would be happy to be contacted by a member of the research team by telephone to discuss the study further.

Patients can be approached at least 7 days prior to their scheduled colonoscopy by the research team. The research team will contact the patient and discuss the study with them in detail.

The consenting process will carefully explore with the eligible patient,

- The nature of the study,
- The additional risks and benefits of including a CCE
- The unlikely possibility of needing a subsequent colonoscopy should significant pathology be missed by the initial colonoscopy.

It is important that participants understand, accept and feel they can:

- swallow the capsule.
- manage the extended fast, additional laxatives (boosters) and logistic burden of the paired CCE and colonoscopy over colonoscopy alone.
- the possibility of having a deferred colonoscopy in the context of having a distant site CCE or unexpectedly not being able to proceed with the same day colonoscopy after swallowing the CCE will also be explored.

Patients may wish to discuss the study with family and/or carers before deciding to participate, but it is imperative that symptomatic patients do not have their primary investigation delayed to accommodate inclusion into the study.

Patients who are happy to take part in the study will be invited to consent. At a minimum the research team must receive verbal consent from the patient to join the ColoCap study, to enable the clinical team to schedule the CCE procedure. This verbal consent must be documented in the patients' medical notes.

Written consent can be received either:

- By post or email: Patients will receive a copy of the consent form and patient information leaflet. The patient can complete the consent form and return it by post or email to the research team or bring the consent form with them on the morning of their procedure. It will be countersigned by a delegated member of the study team as per local site SOP guidance.
- Where the patient posts/emails the consent form back ahead of the day of the procedure, their willingness to continue with the study must be checked when they attend their appointment.
- Face to Face: Patients can consent face to face on the day of their procedure.

7.4 Payment

Participants can claim up to £25 for reimbursement of travel expenses from their hospital. Sites will retrospectively claim this cost from the study team as per the site agreement.

Following the recommendation of our PAG group and participant feedback from our previous CCE patient experience research which highlights that many participants will not feel well enough to perform their usual day-to-day activities, including attending work, participants will also receive £100 for out-of-

pocket expenses. This has been calculated using the London living wage for an average workday (accounting for annual increases). This will be paid for participants who complete or attempt to complete both procedures.

7.6 Data collection

For each recruited participant an eCRF must be completed on REDCap. Clinical and demographic data will be obtained directly from the participants and their medical records.

Contact details for participants that consent to being contacted for interviews (see section 10) will be obtained directly from the participant on the informed consent form. Participating sites must provide the Sponsor with the contact details for these participants via secure NHS Mail to yhs-tr.colocapstudy@nhs.net.

7.6 Withdrawal criteria

If a patient wishes to withdraw from the study, they shall be free to do so at any point with no detriment to their medical care.

If a patient withdraws from the study after they have had all interventions any data collected will be used for the study.

It is possible that the patient will not be able to swallow the colon capsule.

It is possible that despite giving fully informed consent, the participant feels unable to proceed to a same day colonoscopy. That being the case, efforts will be made to offer a next day or deferred colonoscopy. Patients must be advised that a next day colonoscopy would further extend their fast, although they would be encouraged to take clear fluids. A deferred colonoscopy would involve a repeat of the bowel preparation. Nonetheless, some participants may not feel able to proceed to that colonoscopy and will be withdrawn from the study. Any data collected will be used for the study. The CCE report will be available to the site however the patients' onward investigations will be a clinical decision.

The withdrawal of the patient should be documented on the enrolment log. Where a participant provides a reason for withdrawal this should be documented.

A withdrawal eCRF must be completed on REDCap for withdrawn participants.

7.7 End of trial

The end of study is the date of the completion of the data collection for all participants.

The Research Ethics Committee (REC) which gave a favourable opinion of the research will be notified of the end of study, in writing, using the appropriate form within 90 days of the end of the study.

8 TRIAL INTERVENTION: WS1

The trial intervention will be a CCE as an additional procedure prior to the participants colonoscopy. All colon capsules used for the study will be CE marked and are being used for their intended purpose therefore this study falls outside of the medical device legislation.

8.1 Bowel preparation:

The participant will be provided with an instruction sheet detailing the bowel preparation process that should be followed.

The current bowel preparation regime used in the UK for CCE evaluations will be followed for this study. This is similar to standard bowel preparation for colonoscopy. This regimen may vary slightly between hospitals however generally this will be as described below.

A low residue diet will be taken for 3 days and then on the day before the procedure the participant will be asked not to eat any food. Clear liquids only may be consumed throughout that day. The participant will be encouraged to take approximately 8 glasses (240 ml each) of clear liquids. These will help the participant stay hydrated and start cleaning the colon. Examples of clear liquids are water, tea or coffee without milk, clear or fizzy sports drinks, apple juice from concentrate or clear fruit cordials, iced lollies or flavoured jelly, chewing gum or boiled sweets. No red or purple colours should be taken, and broth and soup are not clear liquids. Most medicines can continue to be taken conventionally, however the responsible clinical team will provide formal guidance regarding the omission of certain medicines, in line with existing practice, as necessary.

Beyond these restrictions the participant will continue their day without any restrictions but at 5pm they will need to drink one litre of the strong laxative solution used to cleanse the bowel. From this point the participant will need ready access to a toilet and assistance as necessary. They may need to use it urgently. The laxative will be polyethylene glycol 3350 plus ascorbate (PEG, Moviprep®) and many people find it easier to drink if it is first chilled (or flavoured with a non-blackcurrant cordial). It will need to be prepared beforehand by combining a pair of sachets to one litre of water. The recommended rate is to drink approximately 1 glassful every 15-30 minutes, noting that drinking at too fast a rate could lead to vomiting of the solution. For sites unable to access the current NHS E pilot laxative, Moviprep® from their pharmacies then Plenvu® could be used as an alternative PEG based laxative.[46-47]

The participant will then continue on a clear liquid diet and at 8pm, repeat the steps above and take a second litre of PEG solution. The participant will once again continue with the clear liquid diet over the evening until going to bed. The responsible clinical team will provide guidance about how best to take medicines during the bowel preparation in line with standard care. In general, this involves avoiding medicines in the hour before and after the bowel preparation.

On the morning of the day of the procedure, the participant will need to get up early to ensure that they attend the site where the colon capsule is to be swallowed for 8 am. This will usually be at the endoscopy unit in the local NHS Hospital Trust/Board although it may be a site distant from the endoscopy service.

The participant will be advised about morning medicines by the responsible clinical team, but they would normally be expected to take their medicines first thing with a small amount of water. After this, the participant should not eat or drink anything until advised by the clinical team.

8.2 The CCE procedure: On the day of the procedure, the participant will attend the endoscopy unit or designated clinic early, wearing a loose-fitting top. A process of ‘booking in’ will take place in line with colonoscopy and CCE, which includes safety checks and here, a trained nurse will confirm the participant’s preparedness, fitness and consent to proceed with the CCE and the colonoscopy

combined. There may be some variation between Trusts. These will be discussed and agreed at site set up.

The participant will take 1 or 2mg prucalopride (a prokinetic), allergies excepting, as appropriate. The 2mg dose will be offered to older participants or those in whom reduced gut motility is clinically suspected.[48, 49] Where the participant has provided written consent prior to the day of the procedure they may be directed to take this before arriving at the endoscopy unit.

A recorder belt containing radio sensors will be fitted. The recorder sits around the waist in a pouch with a strap around the shoulder holding it in place.

After 45 minutes the CCE, about the size of a vitamin pill will be swallowed. Once swallowed, the clinical team will check the capsule progress via the recorder to ensure that the equipment is functioning, and that the recorder is wirelessly capturing the images obtained by the capsule. This will take about 30 minutes and thereafter the participant will be free to return home, should they wish. If the participant returns home, they will be given the 'boosters' and the bisacodyl suppository to take with them. The belt and recorder will need to be worn until the colon capsule has passed or until they have to attend for their pre-arranged colonoscopy and the participant will need to be able to follow the procedural instructions to take additional laxatives that act as 'boosters' to help move the capsule through the bowel (see below). The participant will need to have ready access to a toilet and assistance as necessary. They may need to use it urgently. Plenty of clear fluids should be taken throughout the day and participants will be encouraged to remain active during the CCE procedure as activity promotes gut motility. Experience from the UK evaluations is that very few patients suffer pain during the passage of the colon capsule.

Currently the only CCE service widely available in the UK is provided by the PillCam™ COLON capsule, supplied by Medtronic. But other capsules may enter clinical practice in future, such as the OMOM CC (Jinshan company) and the PC-I manufactured by ANKON Technologies Co., Ltd. All licensed CCE services with the technical ability for video upload onto accessible reading platform for review would be eligible for evaluation in this study.

8.3 Additional medicines taken during CCE: Additional medicines, known as 'boosters' are taken during the CCE procedure to facilitate its passage through the intestine. Up to two boosters are taken. A bisacodyl suppository may also be required at the end of the procedure. The CCE recorder generates 'alerts' to prompt the patient to take these additional medicines. Since these need to be timed, the participant will have to be able to understand their purpose and comply with the accompanying instructions. They are generally well tolerated but can cause discomfort and diarrhoea. The 'alerts' sent by the CCE reader may vary depending on the supplier but generally they will follow the format detailed below.

<u>Alert 1:</u> once the capsule has entered the small bowel the participant will receive this alert and should take a booster. This booster will contain 30 ml of the sodium phosphate solution and 50 ml of gastrografin. Since the booster helps propel the capsule into and through the colon, ready and urgent access to a toilet is a necessity. If the participant is returning home the booster should not be taken until they have arrived at home. The participant will continue to take about one litre of water over the next hour, remaining active and wait for the next alert. Participants with an allergy to iodine containing contrast media will be offered a different booster.

The next alert will be either ‘End of Procedure’ or ‘Alert 2’. End of procedure indicates to the patient that the CCE has been excreted.

Alert 2: Three hours after ‘Alert 1’ the participant may receive this alert and should take the second smaller booster. This is 15 ml of sodium phosphate mixed with water and 50 ml of gastrografin. The participant should continue drinking about 0.5 L of water over the next hour and wait for the next alert.

The next alert will either be ‘End of Procedure’ or ‘Alert 3’.

Alert 3: Two hours after ‘Alert 2’ the participant will receive this alert if the capsule has not been excreted. Here the 10 mg bisacodyl suppository should be inserted into the rectum to allow the capsule to be excreted.

End of procedure: This alert will prompt the participant to contact the clinical team and to attend the endoscopy unit for a colonoscopy. At this stage the participant should not eat or drink. The participant can remove the belt and recorded at this stage.

The capsule usually passes in 4-6 hours and based on current experience we anticipate 80-85% of capsules to have passed within 8 hours. The capsule is disposable and can be flushed away safely down the toilet when it passes.

8.4 Colonoscopy: Later in the afternoon the participant will attend the endoscopy unit where a colonoscopy will be performed. If the capsule has not been excreted by 4 pm the participant should still attend the endoscopy unit for a colonoscopy. The belt and recorder will be removed at the endoscopy unit. It is safe to perform the colonoscopy with the CCE still within the bowel. The capsule may be removed during the colonoscopy at the discretion of the endoscopist. It is not anticipated that the colonoscopy will be performed after 6 pm at the discretion of the participant and the endoscopy unit. The participant will need to understand that the colonoscopy will be scheduled for the end of the afternoon, but it is anticipated that some flexibility in timing may be possible without impacting on other non-study patients on the list.

Recovery and patient follow up will follow standard clinical practice. A provisional colonoscopy report is ordinarily provided to the patient immediately after the procedure. This study compares diagnostic accuracy by optical means, although colonoscopic findings may be supported by subsequent histology. The histology from any biopsies or polypectomies will be reconciled when available. The CCE reader will be blinded to the colonoscopy result.

All makes of colonoscopy will be allowed in this study. Colonoscopy is defined as one performed by a trained colonoscopist as the clinically identified primary colorectal diagnostic test for the recruited patient. Key performance indicators of colonoscopy quality and safety exist as part of accreditation for colonoscopy in the UK and these will be recorded for all colonoscopists who take part in this study.

In newer colonoscopes additional optical facilities may be available such as ‘Narrow Band Imaging’, Texture and Color Enhancement Imaging’ and a ‘computer-aided detection application’ that uses artificial intelligence (AI) to increase polyp detection rate (ENDO-AID CADe). The use of these facilities will be recorded if used at the colonoscopy.

It is possible that despite giving fully informed consent, the participant feels unable to proceed to a same day colonoscopy. That being the case, efforts will be made to offer a next day or deferred colonoscopy

(within 2 weeks of the CCE). Nonetheless, some participants may not feel able to proceed to that colonoscopy. Based on a review of the literature we estimate that this may occur in up to 10% of cases.[24.50] Our aim is that by providing potential participants with sufficient information and support this number can be kept to a minimum.

8.5 CCE findings reporting: After the procedure is complete, the research team will supervise the downloading of the data to a secure computer and co-ordinate the clinical analysis of the images into a video for review. Trained and approved CCE readers will record all colorectal findings. Mucosal lesions will be defined by their size, site, extent and characteristics, as appropriate. CCE results will be available within 7 working days.[51, 52] The CCE readers will be masked to the results of the colonoscopy. If the CCE reader becomes unmasked to the colonoscopy prior or during the reading this must be reported to the study as a Protocol breach.

Participants will be informed of the CCE findings in line with standard clinical care at each site.

8.6 Differences from colonoscopy

Every effort has been made to design the study to be as similar as possible to colonoscopy. The bowel preparation is similar however, the diagnostic accuracy requirement of the NIHR HTA commissioning brief 22/168 inevitably imposes at the minimum, an extension of the solid food fast from approximately 24 hours to 32 hours.[53] It also increases the amount of laxative (as boosters) that needs to be taken and extends the length of the procedural day.

8.6.1 The day before the procedure: In most endoscopy units it is standard practice for patients to have a light, modified breakfast on the morning of the day before the procedure and then take no food until after the colonoscopy. Beyond this, the nature of the clear fluids taken, the split dosing and volume of PEG solution regime and the guidance in taking concomitant medicines are the same for CCE and colonoscopy, however the timing of the regime may differ.

8.6.2 The day of the procedure: All the procedural elements of the CCE outlined above are different from colonoscopy. Whilst the colonoscopy *per se* will be the same, its timing in the day will be delayed. The participant will need to extend their fast by up to a further 8 hours to accommodate the CCE prior to the colonoscopy.

8.7 Conclusion of procedures and post procedural follow up: Immediate recovery support and guidance will align with standard care. Ordinarily the optical colonoscopic findings are made available to the patient immediately after the colonoscopy, pending histology and/or further investigations. The findings of the CCE will be communicated to the participant in a pre-agreed fashion and any differences in findings will be explained, discussed and addressed as outlined above and unblinded to the colonoscopist at this stage, as necessary.

Key patient, investigative and clinical outcome data (including adverse events) will be uploaded onto an eCRF. This will include quality standards for both tests.

8.8 CCE retention and adverse events: Fortunately, adverse events are rare. The capsule cannot be swallowed or is vomited up by the patient on 0.2% of occasions. Once the capsule has been swallowed, adverse events, such as pain, occur on <1% of occasions.[39, 54-56] Retention of a capsule is even rarer (3:1000) but would necessitate additional assessment and likely X-rays, such as a plain abdominal X-ray followed by a CT of the abdomen and pelvis, should the retention be confirmed. Retention is defined by the failure of the CCE to be excreted 14 days after swallowing it and is invariably caused by bowel pathology causing an unanticipated stricture. Onward management would be on clinical grounds but is usually dependent upon the identification of that previously undiagnosed stricture usually caused by IBD (Crohn's disease) or CRC. MRI scans cannot be performed on patients until the excretion of the capsule has been formally documented.

The abdominal X-ray will be performed only if there is a clinical suspicion of capsule retained due to obstruction. Any x-ray or CT imaging performed for the study will be approved by the Research Ethics Committee and will be carried out in accordance with each local centre's Ionising Radiation (Medical Exposure) Regulations 2017 [IRMER17] Employers Procedures.

8.9 Repeat colonoscopy for potentially missed colorectal disease: For some, the CCE may identify a polyp ≥6mm, or other clinically significant pathology, that is not seen at the subsequent colonoscopy.[37] This will prompt the consideration for a repeat colonoscopy within 6 weeks, should it be clinically indicated. Here the CCE findings will be unblinded to the colonoscopist and the findings of the repeat procedure will be recorded in the eCRF.

8.10 Options for off- or distant site CCE: In response to attempts to recruit from geographically isolated and more socioeconomically disadvantaged populations we will be exploring options for off- or distant-site CCE.[57] This may require some modification to the study design since it may not be possible to provide same day colonoscopy for all participants. Any modifications will be discussed on a site-by-site basis and agreed with the Sponsor. Here the initial colonoscopy will be scheduled for within 2 weeks after the CCE. The participant and colonoscopist will be blinded to the findings of the CCE at that colonoscopy but, on conclusion of the procedure and the creation of the optical report, the CCE findings will be unblinded. This will permit any clinically significant lesions detected at CCE but missed at colonoscopy to be reviewed by a targeted same-sitting repeat colonoscopy. The findings of that second colonoscopy, should it be required, will be recorded in the eCRF.

9 SAFETY REPORTING

9.1 Definitions

Term	Definition
**Adverse Event (AE)**	Any untoward medical occurrence in a study participant which does not necessarily have a causal relationship with the study treatment or intervention (e.g. abnormal laboratory findings, unfavourable symptoms or diseases) is classed as an adverse event (AE)
**Adverse Reaction (AR)**	An adverse reaction (AR) is any untoward and unintended response in a participant to a product or study procedure where there is evidence or argument to suggest a causal relationship.

	Any adverse event judged by either the reporting investigator or the sponsor as having reasonable causal relationship to a product or study procedure qualifies as an AR
**Serious Adverse Event (SAE)**	An adverse event, adverse reaction, or unexpected adverse reaction is defined as serious if it:<br>(a) results in death,<br>(b) is life-threatening<br>(c) requires hospitalisation or prolongation of existing hospitalisation,<br>(d) results in persistent or significant disability or incapacity, or<br>(e) consists of a congenital anomaly or birth defect<br>(f) is otherwise considered medically significant<br><br>ALL AE/SAEs should be collected for all trial subjects from the commencement of any study related procedures (including screening procedures).
**Serious Adverse Reaction (SAR)**	An adverse event that is both serious and, in the opinion of the reporting Investigator, believed with reasonable probability to be due to one of the trial interventions, based on the information provided.

9.2 Operational definitions for (S)AEs

Any untoward medical occurrence in a study participant which does not necessarily have a causal relationship with the study intervention will be classed as an Adverse Event (AE).

AEs occurring from the point of consent until after the colonoscopy will be documented on the CRF and in the participants medical records. All AEs must be assessed for their relatedness to the study intervention.

An AE will be defined as a Serious Adverse Event (SAE) if it:

(a) results in death,

(b) is life-threatening

(c) requires hospitalisation or prolongation of existing hospitalisation,

(d) results in persistent or significant disability or incapacity, or

(e) consists of a congenital anomaly or birth defect

(f) is otherwise considered medically significant

9.3 Recording and reporting of SAEs

In line with colonoscopy Key Performance Indicators (KPIs) all **SAEs** occurring from the time of **written informed consent** until 30 days post cessation of trial intervention must be recorded on the Research Related SAE/SUSAR Initial Report Form and emailed to the Sponsor (yhs-tr.research.governance@nhs.net) **within 24 hours** of the research staff becoming aware of the event. Once all resulting queries have been resolved, a copy should be retained in the site file.

For each **SAE** the following information will be collected:

- full details in medical terms and case description
- event duration (start and end dates, if applicable)
- action taken.
- outcome
- seriousness criteria
- causality (i.e. relatedness to trial investigation), in the opinion of the investigator
- whether the event would be considered expected. These are:
    - inhalation of the capsule
    - vomiting of the capsule
    - abdominal pain requiring hospital attendance.
    - dehydration/collapse requiring hospital attendance.
    - retention (capsule has not been excreted 14 days after swallowing)
    - rectal bleeding requiring hospital attendance.
    - suspected perforation
    - complications arising from colonoscopic sedation and analgesia.

Any change of condition or other follow-up information should be emailed to the Sponsor as soon as it is available or at least within 24 hours of the information becoming available. Events will be followed up until the event has resolved or a final outcome has been reached.

9.4 Reference Safety Information (RSI)

**Expected Events**	**Procedure**
Unable to swallow the capsule	CCE
Inhalation of the capsule	CCE
Vomiting of the capsule	CCE
Abdominal pain requiring hospital attendance	CCE/ Colonoscopy
Dehydration/collapse requiring hospital attendance	CCE/ Colonoscopy
Capsule retention	CCE

Rectal bleeding requiring hospital attendance	CCE/ Colonoscopy
Suspected perforation/perforation	CCE/ Colonoscopy
Skin irritation	CCE
Complications arising from colonoscopic sedation and analgesia	Colonoscopy

9.5 Responsibilities

Principal Investigator (PI):

Checking for AEs and ARs when participants attend for treatment / follow-up.

1. Using medical judgement in assigning seriousness, causality and expectedness using the Reference Safety Information approved for the trial.
2. Using medical judgement in assigning seriousness and causality and providing an opinion on whether the event/reaction was expected using the Reference Safety Information approved for the trial.
3. Ensuring that all SAEs are recorded and reported to the sponsor within 24 hours of becoming aware of the event and provide further follow-up information as soon as available. Ensuring that SAEs are chased with Sponsor if a record of receipt is not received within 2 working days of initial reporting.
4. Ensuring that AEs and ARs are recorded and reported to the sponsor in line with the requirements of the protocol.
5. Yellow card reporting to report suspected side effects to bowel preparation.

Chief Investigator (CI) / delegate or independent clinical reviewer:

1. Clinical oversight of the safety of patients participating in the trial, including an ongoing review of the risk / benefit.
2. Using medical judgement in assigning the SAEs seriousness, causality and whether the event was anticipated (in line with the Reference Safety Information) where it has not been possible to obtain local medical assessment.
3. Using medical judgement in assigning whether and event/reaction was anticipated or expectedness in line with the Reference Safety Information.
4. Immediate review of all related, unexpected serious adverse events.
5. Review of specific SAEs in accordance with the trial risk assessment and protocol as detailed in the Trial Monitoring Plan.

Sponsor: (NB delegated to CI and ColoCap study team)

1. Central data collection and verification of AEs, ARs, SAEs according to the trial protocol onto a database.
2. Reporting safety information to the CI, delegate or independent clinical reviewer for the ongoing assessment of the risk / benefit according to the Trial Monitoring Plan.

3. Reporting safety information to the independent oversight committees identified for the trial (Data Monitoring Committee (DMC) and / or Trial Steering Committee (TSC)) according to the Trial Monitoring Plan.

Trial Steering Committee (TSC):

In accordance with the Trial Terms of Reference for the TSC, periodically reviewing safety data and liaising with the DMC regarding safety issues.

Data Monitoring Committee (DMC):

In accordance with the Trial Terms of Reference for the DMC, periodically reviewing overall safety data to determine patterns and trends of events, or to identify safety issues, which would not be apparent on an individual case basis.

9.6 Notification of deaths

The PI at each site is responsible for notifying the Sponsor of the death of a study participant with 30 days of colonoscopy.

This report will be immediate (within 24 hours of becoming aware of the death).

9.7 Pregnancy reporting

Pregnancy is not considered an AE unless a negative or consequential outcome is recorded for the mother or child/foetus. If the outcome meets the serious criteria, this would be considered an SAE.

9.8 Reporting urgent safety measures

If any urgent safety measures are taken the CI/Sponsor shall immediately and in any event no later than 3 days from the date the measures are taken, give written notice to the relevant REC of the measures taken and the circumstances giving rise to those measures.

9.9 The type and duration of the follow-up of participants after adverse reactions.

Participants must be followed up until the adverse reaction has resolved or a final outcome has been reached.

10 SUB STUDIES

10.1 Intra and Inter observer sub study.

10.1.1 Background

A prior study has suggested that there is poor agreement on ‘indication for colonoscopy’ after CCE.[29] However, concordance rates published in the literature vary widely and no studies have assessed all the outcomes below in our population of interest.[30]

10.1.2 Aims

To describe the intra- and inter-observer variation of CCE reading to allow further interpretation of our results and how they can be improved. The degree of agreement will dictate the variability with which CCE can reliably prevent the need for colonoscopy.

10.1.3 Objectives

1. Describe the intra- and inter observer variation of the following clinical outcomes:
   - all detected mucosal lesions (CRC, pre-malignant polyps and IBD colitis)
   - suspected CRC
   - number of polyps
   - polyp size (<6, 6-9, >9mm) and site
   - colitis (suspected IBD)
   - bowel preparation adequacy[38]
   - completion.
2. Describe the improvement in accuracy that can be achieved for the above endpoints by double read of colon capsule videos.[58,59]

10.1.4 Design

140 colon capsule videos will be assessed by a second reviewer. An additional 140 colon capsules will be assessed for a second time by the same reviewer. A randomised system will be used to allocate videos to reviewers so that reviewers are unaware as to whether they are reviewing a video for the first or second time to minimise any potential bias. Reviewers will be unaware of any colonoscopic findings when completing second reads. All the clinical observations above will be collected at each review. A prevalence of any mucosal lesion is expected to be 40%. If the agreement kappa is 0.9, a 95% confidence can be expected (one sided) that it is >0.82 with a sample size of 140 (kappa Size R package based on Donner and Rotondi 2010).[45] Agreement kappas will be presented with confidence intervals to describe the clinical outcomes in terms of inter and intra-observer variation.

10.2 SWAT

The Cochrane recruitment and retention reviews (both led from Aberdeen) found very little evidence for strategies targeting under-served groups and the limited evidence available is methodologically poor. Trial Forge, led by ST, has a SWAT Network led by the York Trial Forge Centre that is currently doing a SWAT prioritisation exercise and we expect recruitment and retention of under-served populations to be a SWAT priority. Greater inclusion of under-served populations is also an NIHR EDI strategic priority. We will therefore include two SWATs targeting under-served groups.

We will do two SWATs. Our completed INCLUDE Socioeconomic Disadvantage Framework has highlighted transport has come up as a likely problem for people experiencing socioeconomic disadvantage. We will therefor evaluate offering door-to-door taxis to all potential participants at up to three Colo-Cap sites. We will compare recruitment (i.e., proportion randomised) with recruitment at

other centres and especially that of people experiencing socioeconomic disadvantage as measured using the Index of Multiple Deprivation. The study will not be randomised: we will select only urban test sites with site catchment area that suggests we should see a high proportion of socioeconomically disadvantaged in Colo-Cap. The primary reason for this is the potential cost of a taxi-based intervention, which means we must be selective and cautious to avoid budgetary problems. It is also worth noting that our primary SWAT proposal at budget submission was based on electronic pre-payment cards, which because unfeasible due to changes in the pricing of the cards, withdrawal of support for the card by the University of Aberdeen, and the impact of 'Know your customer' banking laws on the operational feasibility of the cards. We are therefore replacing the electronic pre-payment card SWAT with the taxi SWAT but working within the budget framework of the original SWAT.

We do, however, still propose to do an evaluation linked to the £100 'thank-you' payment in Colo-Cap. All of these payments will now be as vouchers, but it remains unclear what type of voucher is likely to be preferred by potential participants, or whether offering any sort of choice impacts recruitment. We propose therefore to evaluate the uptake of offering a choice of up to four different types of voucher. Again, we do not propose randomisation. Rather, our primary aim is an evaluation of voucher choice by participant demographic, and this does not need a randomised evaluation. The choice of voucher is to be determined, but LoveToShop, which has become a standard within UK universities, will be one of them. We will consider an evaluation of offering vouchers in electronic versus physical form if Colo-Cap central office capacity, and budget, allow.

10.3 Economic Evaluation: models of cost effectiveness.

10.3.1 Overview: This workstream (delivered by YHEC) will estimate the cost-effectiveness of CCE when compared with current diagnostic testing (colonoscopy).

The decision problem considered in this workstream is aligned with the populations that CCE is evaluated in within the proposed study. Therefore, we will develop three health economic models to evaluate the costs and benefits of CCE. The populations considered are people:

a. at risk (FIT adjusted) of CRC: symptomatic population;
b. at risk of IBD colitis and
c. at risk of CRC: surveillance population post polypectomy.

The intervention is defined as CCE, the tested diagnostic, with follow up colonoscopy as required based on the pathology identified or the incompleteness/inadequacy of the CCE examination. The comparator is colonoscopy alone, the current standard of care for the diagnosis of CRC, polyps and IBD colitis. Data on sensitivity and specificity of CCE will be used to inform downstream pathways. Further details on the economic modelling of CCE for the three populations are described below.

10.3.2 CRC models (surveillance and symptomatic; populations a. and c.)

The proposed study explores the diagnostic accuracy of CCE as a comparator to colonoscopy for detection of CRC. Therefore, the potential impacts of CCE, within this cancer indication, are likely to be resource change in the short term from reduced colonoscopy procedures and differences in costs and outcomes in the long-term from changes in health outcomes.

Health outcomes in the long term are anticipated to be affected by the identification and subsequent colonoscopic removal of pre-cancerous polyps within the population considered. The removal of pre-cancerous polyps may result in a lower incidence of CRC later in life. Therefore, the economic models for the surveillance and symptomatic populations will be developed to appropriately capture this.

Two models will be developed for the surveillance and symptomatic populations. Both models will have the same conceptual structure: there will be two components, one capturing the diagnostic pathway and another component to capture the CRC progression.

We propose building these two models using a patient-level simulation (PLS) approach based on discrete health states. The outcomes at the end of the diagnostic pathway will be used to determine onward management and risk of progression. The diagnostic component will be specific to each population to capture the unique follow-up protocols in place. The natural history of CRC will be captured through discrete health states consisting of no polyps; low-risk; high-risk polyps; CRC; post-CRC; and death.

The risk categorisations will be based on the number and size of pre-cancerous polyps as determined by national guidelines. The risk of polyp recurrences post-polypectomy is variable depending on the risk status of the individual at the primary polypectomy. Additionally, the follow-up and routine testing from surveillance and screening protocols are time dependent and specific for the assigned risk category of the individual. Therefore, a PLS is applied here to appropriately capture the different pathways by which people can travel through their life. A PLS model includes memory of previous screening and test results on a per patient basis, which is not possible in a cohort-based Markov or decision tree model structure. This will allow flexibility to include the variation in development of CRC and pre-cancerous polyps within these populations.

Resources used will be measured in both economic models and the relevant costs applied to them. These include diagnostic tests; biopsies; adverse events; and cancer treatment.

Costs will be estimated from the perspective of the NHS and Personal Social Services. Utilities will be applied to each of the health states in the model to estimate quality-adjusted life years for each patient simulated. Life-years and other event counts of interest (such as total colonoscopies) will also be estimated.

The key outputs of the models will be the total incremental costs, total incremental QALYs and life-years, cost per colonoscopy avoided, and the excess number of CRC detected.

There will be limitations to the data used to inform the models. Therefore, parameter uncertainty will be tested within the models through multivariate deterministic and probabilistic sensitivity analysis. The results of these analyses will be presented numerically and graphically.

10.3.5 IBD model (population b.)

The third model will be developed to capture the economic impact of using CCE within the diagnostic pathway for IBD colitis. All relevant resource use and costs will be captured within the model. As above, the results will include total incremental costs as well various sensitivity analysis around key parameters. We propose building a decision tree structure to capture the diagnostic pathway for people presenting with IBD colitis with a short, one-year time horizon to capture the immediate impact of introducing CCE within this pathway. This is based on previous models that have been published in literature.[32, 33]
The evaluation of cost effectiveness: will be completed by month 40.

10.4 Qualitative evaluation of patient and clinician experience: workstream 3

Workstream 3 will be carried out by researchers at the University of York.[61-63]This workstream is an embedded qualitative project exploring patient preference and acceptability, with a focus on what a CCE diagnostic service should look like from a patient perspective, in a way able to connect to the "messy realities" of practice. Building on existing pilot work, this 18-month qualitative evaluation engages with patient and clinician experience of using CCE as a diagnostic tool. Our aim is to be inclusive and engage with the perspectives of patients who may experience particularly difficulties in accessing care. We will be especially sensitive to the experiences of those traditionally excluded by research and this explains why our approach is preferred to a questionnaire.

In workstream 3 there will be in-depth on-line or telephone interviews with 35 participants which will explore the experiences of patients who have been offered CCE. Topics include initial responses, when offered CCE; the influence of others (including health care professionals, family members and peers) on their decision to accept CCE; their expectations and experiences of the process, including what went well and what could be improved; and their perceived value of CCE. Participants will be purposely selected and include a range of ages, ethnicities and socio-economic backgrounds, (potential) disease groups and outcomes, and a mix of those living in urban and rural settings (including those who may struggle to access secondary care). Consistent with involve guidelines, patients will receive a payment of £30 for participating in the research.

Additionally, the researchers from the University of York will conduct interviews with 20 clinicians drawn from different parts of the UK who will be selected according to different degrees of enthusiasm for CCE diagnostics. Interviews will first, explore care professionals experience of negotiating the use of CCE, including identifying potential patient barriers/facilitators and second, discuss their response to the findings exploring patient experience.

These patients and clinicians will be asked to provide written consent to the interviews which will be conducted remotely by a researcher at the University of York. The interviews will be audio recorded, transcribed by an external transcriber and anonymised before analysis. Analysis will identify themes, which are first interrogated as a means of understanding a particular case; and second, compared across cases. Our aim is to move beyond a descriptive account and provide a thematic account able to inform optimal care, consistent with patient preferences. In presenting our analysis we will further contextualise the experience of our sample by comparing it to the experience of those using colonoscopy, as established by the literature.

Output: Our aim is to move beyond a descriptive account and provide a thematic account, using patient and clinician experience, to help understand the findings from WS1 & WS2. In presenting our analysis we will further contextualise the experience of our sample by comparing it to the experience of those using colonoscopy, as established by the literature.

Timeline:
Months 15-17: Refine research instruments, identify and generate patient sample.
Months 18-24: Conduct patient interviews, undertake preliminary analysis for use in interviews with clinicians and identify sample of clinicians.
Months 25-27: Conduct interviews with clinicians.
Months 28-32: Analyse and synthesis material; writing up findings and produce guidance.

11 STATISTICS AND DATA ANALYSIS

11.1 Sample size aim

A sample size calculation was developed to achieve the primary outcome of the diagnostic accuracy of CCE compared to colonoscopy. We will need completely examined and adequately prepared CCE and colonoscopy diagnoses from 657 people. We anticipate 10% of participants that agree to take part will subsequently decline one or other of CCE and colonoscopy (as seen in comparable European studies). [22,23, 24, 50] Also, we anticipate that in 25% cases where both CCE and colonoscopy are achieved, results of one or other of the tests will be incomplete or inadequately prepared. Therefore, to get 657 paired test results we expect that we will need to consent 973 participants.

657/.9/.75 = 973 participants to be recruited to the study

11.2 Sample size calculation:

The sample size calculation is primarily driven by the requirements to test the sensitivity of CCE compared to colonoscopy. We followed the approach recommended by Chu and Cole (2007) because the expected sensitivity is high.[64] The statistical test that is used is a test of a single-sample proportion using an exact binomial test:

H0: p = p0 versus Ha: p > p0

Where p0 is the null sensitivity proportion we wish to rule out, set to 0.90. Following Chu and Cole, for 90% power, a one-sided 5% alpha, and an expected sensitivity of 0.95 requires 263 disease positive cases. 263 cases provide 93% power, but due to the sawtooth nature (i.e., non-monotonic) of the power function for an exact test of a single proportion, Chu and Cole recommend taking the lowest sample size N such the required power (here 90%) is guaranteed for sample sizes larger than N. The Stata code block, below, plots power for sample sizes for the above test between 250 and 275 to show this. The R function 'power.diagnostic.test' in the package 'MKmisc' can also be used to derive this.

*** Stata code block begins here ***

```
power oneprop 0.90 0.95', n(250(1)275) test(binomial) critvalues onesided /// table(, formats(alpha_a
"%7.3f"
power "%7.3f")) /// graph(yline(0.9) plotopts(mlabel(N)))
*** Stata code block ends here ***
```

We expect a colorectal disease prevalence of 40% in the cohort recruited.[41, 54] Using the formula N_controls = N_cases [(1-Prev)/Prev] where N_cases is the required number of cases and Prev is the expected prevalence we derive the number of disease negative controls as 394 and a total sample size of 263 + 394 = 657. 394 disease negative controls give above 90% power to rule out 75% specificity given an expected specificity of 85%.

11.3 Statistical analysis

There will be one final statistical analysis when recruitment is complete, and the study database is cleaned, checked and locked. Only participants with both a complete and adequately prepared CCE and colonoscopy will be included in the primary analysis to be used to determine accuracy statistics.

The intervention is a CCE and the reference test is colonoscopy. A test positive result will be the detection of mucosal lesions (CRC, polyps or colitis), at the patient level, by CCE; the colorectal disease positive status will be the detection of any mucosal lesion at the patient level, by colonoscopy. The index test accuracy (its sensitivity and specificity) will be assessed in the first instance based upon a one-sided comparison between the results of the intervention and those of the reference standard. The statistical test is one-sided because this approach assumes that any discrepancy is an error in the index test. This reflects the commission brief specifications.[53] The proportion of participants with inconclusive results, due to an incomplete study and/or inadequate bowel preparation, will be reported and their impact on estimates of test accuracy will be assessed by including them as either test positives or test negatives in the sensitivity analyses.

We know that colonoscopy diagnosis is not 100% accurate and ignoring this could underestimate the value of CCE.[24] To account for this, we will compare CCE findings with the final diagnosis and also use correction methods from Umemneku, Chikere et al’s review of methodology to deal with diagnostic tests in the absence of a gold standard.[65] Final diagnosis of identified mucosal lesions will be informed by relevant histology and a possible second colonoscopy. We will also report specificity, sensitivity, positive and negative predictive values, and likelihood ratios along with their two-sided 95% confidence intervals from this analysis.

Secondary outcomes results will also be analysed and reported separately by specific lesion types and size, on a per-lesion matching basis, on the three patient groups (including FIT) and on other disaggregated groups based on CCE performance characteristics such as age and sex. Other outcomes to be reported are completion and bowel preparation adequacy rates and adverse events. All outcomes will be reported by indication.

11.4 Planned recruitment rate

The recruitment projection is based on an estimate of 30 active centres contributing 2 participants per month over 24 months (4 eligible patients per month; 50% willing to be recruited). A recruitment projection is shown below (Figure 1).

Figure 1: Recruitment projection

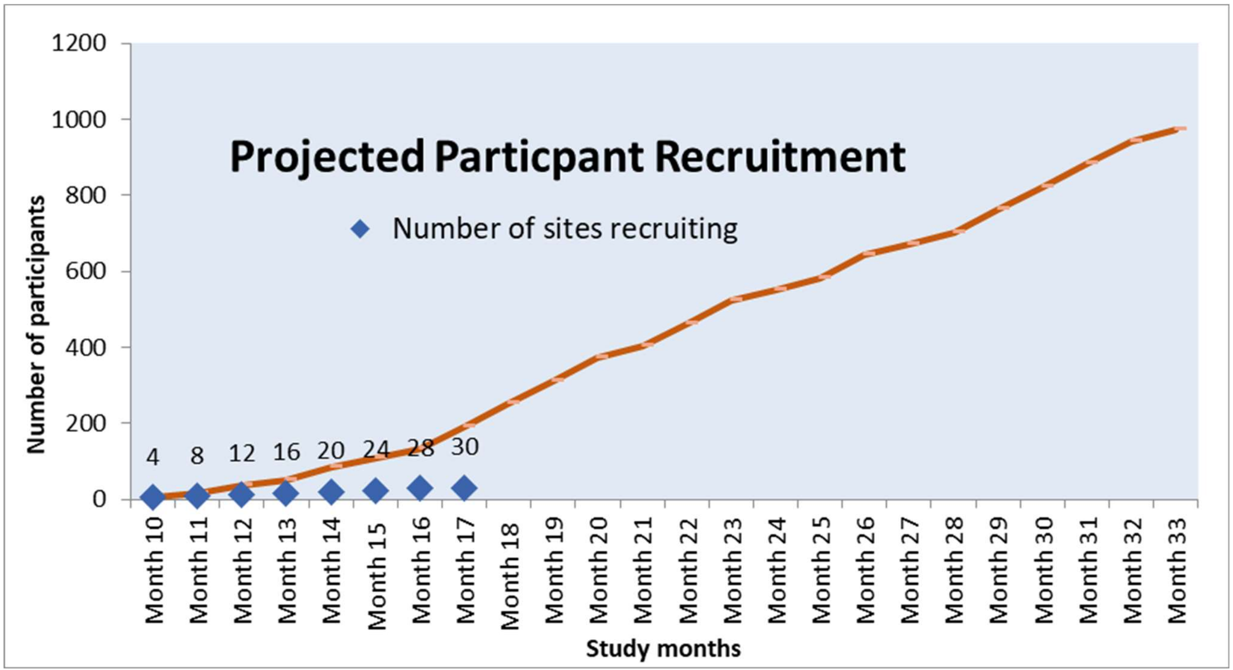

Internal pilot

An internal pilot stage has been included in the study. This 9-month pilot stage will commence in January 2025 (study month 10) and run to end September 2025 (study month 18). Recruitment of 60% of the anticipated number of recruited participants at 9 months will be used to inform progression from pilot phase to full trial.

Figure 2: Stop/go criteria at 9 months.

	Red	Amber	Green
**Centre recruitment**	<60%<br>(<18 centres)	60-99%<br>(≥18 centres)	≥100%<br>(30 centres)
**Participant recruitment**	<60%<br>(<151 pts)	60-100%<br>(≥151 pts)	100%<br>(≥252 pts)
**Action**	Discuss urgently with the TSC and potentially the funder, considering all options including discontinuation.	Consider recruitment strategies and blockages (if centres are not open), including trouble shooting, revised training and support, open additional centres	Proceed seamlessly whist considering recruitment strategies

11.5 Statistical analysis plan

Full statistical analysis plans will be produced prior to any formal analysis being performed on the data.

12 DATA MANAGEMENT

A Data Management Plan is in place and has been approved by the study CI.

12.1 Data collection tools and source document identification

The Source data for this study will be the patient medical records (e.g. letter, clinic notes, laboratory results), capsule software and patient and clinician interview audio and transcripts.

For each participant the research team will complete electronic Case Report Forms (eCRFs). This data will be found in the participants medical records. REDCap will be the eCRF used for this study and will be managed and maintained by York and Scarborough Teaching Hospitals NHS Foundation Trust. A link and login details to REDCap will be provided at time of site opening.

Access to the eCRF will be restricted. At recruiting centres, only authorised personnel will be able to see or make entries or amendments to that site's patients' data.

12.2 Access to Data

Direct access will be granted to authorised representatives from the Sponsor, host institution and the regulatory authorities to permit trial-related monitoring, audits and inspections- in line with participant consent.

12.3 Archiving

Once the study has ended, sites will be asked to complete an end of study checklist. Once this has been returned the Sponsor will confirm that the study documents can be archived. Study documents will be archived by the participating sites.

Study documents will be archived for 5 years from the end of study as per the Sponsors standard operating procedures. After the agreed retention time the Sponsor will authorise destruction of study documents.

13 MONITORING, AUDIT & INSPECTION

A Trial Monitoring Plan will be developed by the TMG, and CIs based on the trial risk assessment. The Sponsor and CI will sign off monitoring plan.

14 ETHICAL AND REGULATORY CONSIDERATIONS

14.1 Research Ethics Committee (REC) review & reports

- Before the start of the study, approval will be sought from a REC for the study protocol, informed consent forms and cover letters.
- Substantial amendments that require review by the REC will not be implemented until the REC grants a favourable opinion for the study.
- All correspondence with the REC will be retained in the Study Master File/Investigator Site File.
- The Chief Investigator will notify the REC of the end of the study.
- If the study is ended prematurely, the Chief Investigator will notify the REC, including the reasons for the premature termination.
- Within one year after the end of the study, the Chief Investigator will submit a final report with the results, including any publications/abstracts, to the REC.

14.2 Peer review

As part of the funding process, the NIHR sought external reviews on the study proposal, which resulted in the study being funded.

The study was peer reviewed by the R&D Group at York and Scarborough Teaching Hospitals NHS Foundation Tust and the Study Adoption Group at Cardiff University.

14.3 Public and Patient Involvement

This study has been shaped by extensive Patient and Public Involvement. One of the co applicants on the funding application is a member of York and Scarborough Teaching Hospitals Public Contributor Group (PCG). They participated in applicants' meetings for stage 1 & 2 development, reviewing and commenting from a patient perspective. A Patient Advisory Group (PAG) was established from the stage 1 focus group. Individuals who have had, or been carers of those with, bowel disease or who have had bowel investigations along with patient representatives on the NHS England CCE Expert Advisory joined the PAG.

Workshops were held in the Sumer of 2023 so that the PAG could review the HTA Funding Committee feedback they had provided for stage 1, to discuss the comments of the HTA Funding Committee and to review the documentation and study design that was being developed for stage 2 of the funding application.

The PAG is now an established group that includes both experienced members and new members with whom we have built up a strong and effective relationship. It will, however, evolve through the lifetime of the trial. It is our plan to also recruit new members from users who have undergone CCE or colonoscopy across the UK. The composition of the group will then better reflect the range of sociodemographic characteristics of people eligible for CCE and colonoscopy.

In support of WS3 we will try to recruit participants of the NHS England CCE pilot patient experience study. We anticipate that there will be 13 (usually remote) PAG meetings during the trial to ensure involvement in key decisions such as the overarching trial set-up, recruitment, study materials production, data collection, analysis and sharing findings. The PAG will also have representation on the TMG and the TSC. Our PPI co-applicant will chair the PAG, with mentoring support as needed.

The PAG will help develop all patient facing materials, including co-producing the patient interview topic guides, patient information sheets and the patient questionnaires in WS3. The PAG will be involved in

identifying patient-centred themes from interviews in WS 3. Those who wish involvement in the analysis of study data will be given relevant training by team members.

The PAG will also provide a patient perspective on the write-up of papers, help produce lay summaries, and assist in presenting the study findings results at conferences, support groups, charity events and online. They will be integral to the dissemination and implementation phase of the programme. PAG colleagues will receive payment for their time in line with NIHR INVOLVE recommendations.

14.4 Regulatory Compliance

The study will not commence until a HRA approval and Favourable REC opinion has been received.

Before any site can enrol patients into the study, the Chief Investigator/Principal Investigator or designee will ensure that appropriate approvals from participating organisations are in place. Specific arrangements on how to gain approval from participating organisations are in place and comply with the relevant guidance.

For any amendment to the study, the Chief Investigator or designee, in agreement with the sponsor will submit information to the appropriate body in order for them to issue approval for the amendment. The Chief Investigator or designee will work with sites (R&D departments at NHS sites as well as the study delivery team) so they can put the necessary arrangements in place to implement the amendment to confirm their support for the study as amended.

14.5 Protocol compliance

Protocol non-compliances are departures from the approved protocol.

Breaches can be serious or non-serious in nature. Not every deviation from the protocol represents a serious breach that must be reported to the Sponsor or regulatory authorities – the majority are technical deviations that do not result in harm to the study subjects or significantly affect the scientific value of the reported results of the study. Breaches of this type, while they must be documented, are not serious breaches or reportable.

Prospective, planned deviations or waivers to the protocol are not allowed unless agreed in writing with the Sponsor in advance.

14.6 Documentation of ALL Breaches

All protocol deviations must be clearly and systematically documented, for appropriate corrective and preventative. At a minimum these should recorded on the Protocol/GCP Deviations Log (R&D/F119) and in an explanatory file note (if appropriate).

Documentation of the breach should include as a minimum:

1. Full details of the breach
2. The date and time of its occurrence
3. Any remedial action undertaken.
4. Assessment by the CI or PI (or delegated individual) as to whether the breach is serious (include signature, date and time)

Breaches and associated documentation will be reviewed during monitoring visits.

14.6 Notification of Serious Breaches to GCP and/or the protocol

A “serious breach” is a breach which is likely to effect to a significant degree –

(a) the safety or physical or mental integrity of the participants of the trial; or
(b) the scientific value of the trial

The sponsor must be notified immediately (and within 24 hours of the breach being identified) of any case where the above definition applies during the trial conduct phase. Notification must be made via email using the ‘Suspected Serious Breach Notification to Sponsor Form’ to yhs-tr.research.governance@nhs.net.

A Sponsor representative (or delegated other) will acknowledge receipt of the breach by noon of the next working day. Acknowledgement will be emailed to the individual who sent the breach unless an alternative method of acknowledgement has been agreed with the Study Team in writing. It is the responsibility of the reporting individual to contact the Sponsor immediately if no acknowledgement is received.

The sponsor will notify the REC that approved the study in writing of any serious breach of

(a) the conditions and principles of GCP in connection with that trial; or
(b) the protocol relating to that trial, as amended from time to time.

14.7 Data protection and patient confidentiality

All investigators and trial site staff will comply with the requirements of the Data Protection Act 2018 and GDPR with regards to the collection, storage, processing and disclosure of personal information and will uphold the Act’s core principles.

- Participants will be allocated a unique Study ID number upon recruitment to the study. This Study ID will be used to identify the participant for the duration of the study.
- Anonymised clinical data will be entered onto the eCRF (Redcap).
- Identifiable data will only be sent to the Sponsor by the sites where the participant has consented to this. Identifiable data will only ever be sent using secure NHS mail.
- The Study Master File will be held on a trust password protected computer or university server or in a locked filing cabinet.
- Full confidentiality will be maintained in accordance with UK law.

14.8 Financial and other competing interests for the chief investigator, PIs at each site and committee members for the overall trial management

Any competing interests that might influence trial design, conduct, or reporting will be collected and documented.

14.9 Indemnity

1. NHS indemnity applies to meet the potential legal liability of the sponsor(s) for harm to participants arising from the management of the research.
2. NHS indemnity applies to meet the potential legal liability of the sponsor(s) or employer(s) for harm to participants arising from the design of the research.
3. NHS indemnity scheme or professional indemnity will apply to meet the potential legal liability of investigators/collaborators arising from harm to participants in the conduct of the research.
4. The equipment required for the study is used as part of standard care. NHS indemnity applies to meet the potential legal liability arising in relation to the equipment (e.g., loss, damage, maintenance responsibilities for the equipment itself, harm to participants or site staff arising from the use of the equipment)

14.10 Amendments

Amendments to trial documentation will follow the regulatory process as per current guidelines and timescales. Amendments will be implemented after Ethical, national co-ordinator and local R&D have provided required approvals.

14.11 Access to the final trial dataset

Anonymised, non-identifiable data will be made available to other researchers, on request and if approved by the Sponsor (on the advice of the TMG), in accordance with the good practice principles for sharing individual participant data from publicly funded clinical trials.[66]

15 DISSEMINATION POLICY

15.1 Dissemination policy

This study has been designed, from the outset, to produce useful, timely and relevant research findings that will allow the rapid implementation of a diagnostic CCE service throughout the UK in support of colorectal disease diagnostics. We have involved the commissioners in all three participating nations during the design of ColoCap and we will communicate with them throughout the course of the study. Through these partnerships we anticipate providing evidence for actionable findings of immediate utility to decision-makers and service users. The main outputs from our research will be presented in peer reviewed international journals and disseminated proactively to NHS England, Scotland and Wales and to Health and Social Care in Northern Ireland within a delivery framework. Working with our PAG and Integrated Care Boards (ICB) we will organise dissemination events aimed at patients and the public.

15.2 Authorship eligibility guidelines and any intended use of professional writers

Any individual who has made a significant contribution to the project will be included in the list of authors, consistent with ICMJE guidelines on authorship.

16 REFERENCES

1. Rutter MD, Brookes M, Lee TJ et al. Impact of the COVID-19 pandemic on UK endoscopic activity and cancer detection: a National Endoscopy Database Analysis. Gut. 2021 Mar;70(3):537-543. doi: 10.1136/gutjnl-2020-322179.
2. Hamilton W, Walter FM, Rubin G, Neal RD. Improving early diagnosis of symptomatic cancer. Nat Rev Clin Oncol. 2016;13(12):740-749. doi:10.1038/nrclinonc.2016.109.
3. Rees CJ, Thomas Gibson S, Rutter MD, et al. UK key performance indicators and quality assurance standards for colonoscopy. Gut. 2016;65(12):1923-1929. doi:10.1136/gutjnl-2016-312044.
4. Rutter MD, East J, Rees CJ et al. BSG/ACPGBI/PHE post-polypectomy and post-colorectal cancer resection surveillance guidelines. Gut 2020; 69:201–223 doi:10.1136/gutjnl-2019-319858.
5. Gavin DR, Valori RM, Anderson JT, Donnelly MT, Williams JG, Swarbrick ET. The national colonoscopy audit: a nationwide assessment of the quality and safety of colonoscopy in the UK. Gut. 2013;62(2):242- 249. doi:10.1136/gutjnl-2011-301848.
6. Derbyshire E, Hungin P, Nickerson C et al. Colonoscopic perforations in the English NHS Bowel Cancer Screening Programme Endoscopy. 2018 Sep;50(9):861-870. doi: 10.1055/a-0584-7138.
7. Ball AJ, Rees CJ, Corfe BM et al. Sedation practice and comfort during colonoscopy: lessons learnt from a national screening programme. Eur J Gastroenterol Hepatol. 2015 Jun;27(6):741-6. doi:10.1097/MEG.0000000000000360.
8. Bjørsum-Meyer T, Toth E, Koulaouzidis A. Carbon footprint from superfluous colonoscopies: potentialities to scale down the impact. Gut. 2021 doi:10.1136/gutjnl-2021-326587.
9. National Institute for Health and Care Excellence (NICE). Computed tomographic colonography (virtual colonoscopy). Interventional procedures guidance. 2005. https://www.nice.org.uk/guidance/ipg129.
10. Spada C, Hassan C, Bellini D et al Imaging alternatives to colonoscopy: CT colonography and colon capsule. ESGE and ESGAR Guideline – Update 2020 Endoscopy 2020; 52: 1022–1036 doi.org/10.1055/a-1258-4819.
11. Shenbagaraj L, Thomas-Gibson S, Stebbing J, et al. Endoscopy in 2017: a national survey of practice in the UK. Frontline Gastroenterology 2019;10:7–15. doi:10.1136/flgastro-2018-100970.
12. Sud A, Torr B, Jones ME, et al. Effect of delays in the 2-week-wait cancer referral pathway during the COVID-19 pandemic on cancer survival in the UK: a modelling study. Lancet Oncol. 2020;21(8):1035-1044. doi:10.1016/S1470-2045(20)30392-2.
13. https://www.england.nhs.uk/statistics/statistical-work-areas/cancer-waiting-times/

14. https://www.nice.org.uk/news/article/nice-recommends-extending-use-of-tests-for-colorectal-cancer-which-couldreduce-waiting-time-for-colonoscopies.
15. Ravindran S, Thomas-Gibson S, Bano M et al. National census of UK endoscopy services 2021. Frontline Gastroenterol. 2022 May 9;13(6):463-470. doi: 10.1136/flgastro-2022-102157.
16. Morris E, Goldacre R, Spata E et al. Impact of the COVID-19 pandemic on the detection and management of colorectal cancer in England: a population-based study. Lancet Gastroenterol Hepatol. 2021 Mar;6(3):199-208.doi: 10.1016/S2468-1253(21)00005-4.
17. COVIDSurg Collaborative The impact of surgical delay on resectability of colorectal cancer: An international prospective cohort study Colorectal Dis. 2022 Mar 14;24(6):708-726. doi: 10.1111/codi.16117.
18. Public Health Scotland. Diagnostic Waiting Times – Quarter Ending: 31 December 2022; 2023.
19. Ismail M, Murphy G, Semenov S. Comparing CCCE to colonoscopy; a symptomatic patient's perspective BMC Gastroenterol. 2022 Jan 24;22(1):31. doi: 10.1186/s12876-021-02081-0.
20. Deding U, Cortegoso Valdivia P, Koulaouzidis A, et al. Patient-Reported Outcomes and Preferences for Colon Capsule Endoscopy and Colonoscopy: A Systematic Review with Meta-Analysis. Diagnostics (Basel) 2021;11(9) doi: 10.3390/diagnostics11091730.
21. MacLeod C, Monaghan E Banerjee A et al. Colon capsule endoscopy Surgeon. 2020 Aug;18(4):251-256. doi:10.1016/j.surge.2020.01.008.
22. Voska M, Zavoral M, Grega T et al. Accuracy of Colon Capsule Endoscopy for Colorectal Neoplasia Detection in Individuals Referred for a Screening Colonoscopy. Gastroenterol Res Pract. 2019 Sep. doi:10.1155/2019/5975438.
23. Pecere S, Senore C, Hassan C et al. Accuracy of colon capsule endoscopy for advanced neoplasia. Gastrointest Endosc 2020; 91: 406 doi.org/10.1016/j.gie.2019.09.041.
24. Kobaek-Larsen M, Kroijer R, Dyrvig A-K et al Back-to-back colon capsule endoscopy and optical colonoscopy in colorectal cancer screening individuals Colorectal Dis. 2018 Jun;20(6):479-485. doi: 10.1111/codi.13965.
25. https://www.nice.org.uk/guidance/dg56
26. https://www.nice.org.uk/guidance/dg11
27. Halder W, Laskaratos FM, El-Mileik H, et al. Review: Colon Capsule Endoscopy in Inflammatory Bowel Disease. Diagnostics (Basel) 2022;12(1) doi: 10.3390/diagnostics12010149.
28. Shi HY, Chan FKL, Higashimori A, et al. A prospective study on second-generation colon capsule endoscopy to detect mucosal lesions and disease activity in ulcerative colitis. Gastrointest Endosc 2017;86(6):1139-46.e6. doi:10.1016/j.gie.2017.07.007.
29. Buijs M, Kroijer R, Kobaek-Larsen M et al. Intra and inter-observer agreement on polyp detection in colon capsule endoscopy evaluations. United European Gastroenterol J. 2018 Dec; 6(10): 1563–1568. doi: 10.1177/2050640618798182.
30. Cortegoso Valdivia P, Deding U, Bjørsum-Meyer T et al. Inter/Intra-Observer Agreement in Video-Capsule Endoscopy: Are We Getting It All Wrong? A Systematic Review and Meta-Analysis Diagnostics; 2022 Oct 2;12(10):2400. doi: 10.3390/diagnostics12102400.
31. Schelde-Olesen B, Bjorsum-Meyer T, Koulaouzidis A et al. Interobserver agreement on landmark and flexure identification in colon capsule endoscopy. Techniques in Coloproctology 2023 Apr 10. doi: 10.1007/s10151-023-02789-z.

32. Turvill J, Turnock D, Holmes H, et al. Evaluation of the clinical and cost-effectiveness of the York Faecal Calprotectin Care Pathway. Frontline Gastroenterol. 2018 Oct;9(4):285-294. doi: 10.1136/flgastro-2018-100962.
33. Zhang W, Wong CH, Chavannes M, et al Cost-effectiveness of faecal calprotectin used in primary care in the diagnosis of inflammatory bowel disease BMJ Open 2019;9:e027043. doi: 10.1136/bmjopen-2018-027043.
34. Bond S, Kyfonidis C, Jamieson M et al Evaluation of an Innovative Colon Capsule Endoscopy Service in Scotland from the Perspective of Patients: Mixed Methods Study Journal of Medical Internet Research; 2023 Apr14;25:e45181. doi: 10.2196/45181.
35. Sulbaran M, Bustamante-Lopez L, Bernardo W et al. Systematic review and meta-analysis of colon capsule endoscopy accuracy for colorectal cancer screening. An alternative during the Covid-19 pandemic? J Med Screen. 2022 Sep;29(3):148-155. Doi.org/10.1177/09691413221074803.
36. Ali H, Pamarthy R, Sarfraz S, et al. Diagnostic Accuracy for Per-Patient Polyp Detection of Second-Generation Capsule Endoscopy Compared to Colonoscopy: A Meta-Analysis of Multicenter Studies. Cureus 2021;13(8):e17560. doi: 10.7759/cureus.17560.
37. Spada C, Hassan C, Galmiche JP et al Colon capsule endoscopy: ESGE Guideline. Endoscopy 2012; 44: 527– 536 doi.org/10.1055/s-0031-1291717.
38. Magalhães R, Arieira C, Carvalho P et al. Colon Capsule CLEansing Assessment and Report (CC-CLEAR): a new approach for evaluation of the quality of bowel preparation in capsule colonoscopy. Gastrointest Endosc. 2021 Jan;93(1):212-223. doi: 10.1016/j.gie.2020.05.062.
39. Cortegoso Valdivia P, Skonieczna-Żydecka K, Elosua A, et al. Indications, Detection, Completion and Retention Rates of Capsule Endoscopy in Two Decades of Use: A Systematic Review and Meta-Analysis. Diagnostics (Basel) 2022;12(5) doi: 10.3390/diagnostics12051105.
40. https://www.nhsinform.scot/tests-and-treatments/non-surgical-procedures/colon-capsule-endoscopy.
41. Turvill JL, McAlindon ME. Interim update on the NHS England colon capsule endoscopy evaluation. Gut. https://gut.bmj.com/content/72/Suppl_2/A40.1 http://dx.doi.org/10.1136/gutjnl-2023-BSG.63.
42. NHS England. NHS rolls out capsule cameras to test for cancer. https://www.england.nhs.uk/2021/03/nhs-rollsout-capsule-cameras-to-test-for-cancer/.
43. Ferreira A I, Lima Capela T, Freitas M et al Colon capsule endoscopy: Predictors of inadequate bowel preparation. United European Gastroenterology Journal. Conference: 30th United European Gastroenterology Week, UEG Week 2022. 10 (Supplement 8) (pp 1048-1049).
44. Gimeno-Garcia A, Gonzalez-Suarez B, Ganzo Z et al Predictive factors for inadequate bowel cleansing in colon capsule endoscopy. Gastroenterol Hepatol. 2022 Oct;45(8):605-613. doi: 10.1016/j.gastrohep.2022.01.003.
45. Macedo Silva V, Lima Capela T, Freitas M et al. Stepping it up: Physical activity is associated with a lower incidence of prolonged gastric transit time in capsule endoscopy Journal of Gastroenterology & Hepatology; 2023 Mar;38(3):404-409. doi: 10.1111/jgh.16074.
46. Deding U, Jensen SS, Schelde-Olesen B et al. Castor Oil in Bowel Preparation Regimens for Colon Capsule Endoscopy: A Systematic Review with Meta-Analysis. Diagnostics; 2022 Nov 15;12(11):2795. doi: 10.3390/diagnostics12112795.

47. Fernandez-Urien I, Eyal I, Schwartz, N et al. Improved low-volume preparation regimen for CCE - a feasibility study. Gastrointestinal Endoscopy. DDW 2023. 97(6 Supplement) (pp AB1253-AB1254).
48. Deding U, Kaalby L, Baatrup G et al. The Effect of Prucalopride on the Completion Rate and Polyp Detection Rate of Colon Capsule Endoscopies Clin Epidemiol. 2022; 14: 437–444. doi: 10.2147/CLEP.S353527.
49. Pillay L, Esan O, Petre A-G et al Does Prucalopride improve outcomes in colon capsule endoscopy? Gut; 72 (Suppl 2), pp. A170-A170 dx.doi.org/10.1136/gutjnl-2023-BSG.294.
50. Vuik F, Nieuwenburg S, Moen S, et al. Colon capsule endoscopy in colorectal cancer screening: a systematic review. Endoscopy 2021;53(8):815-24. doi:https://dx.doi.org/10.1055/a-1308-1297.
51. Rex DK, Rabinovitz R. Variable interpretation of polyp size by using open forceps by experienced colonoscopists. Gastrointest Endosc 2014 Mar;79(3):402-7. doi: 10.1016/j.gie.2013.08.030.
52. Blanes-Vidal V, Nadimi E, Buijs M et al. Capsule endoscopy vs. colonoscopy vs. histopathology in colorectal cancer screening: matched analyses of polyp size, morphology, and location estimates. Int J Colorectal Dis. 2018 Sep;33(9):1309-1312. doi: 10.1007/s00384-018-3064-0.
53. https://www.nihr.ac.uk/documents/22168-the-diagnostic-accuracy-of-colon-capsule-endoscopy-commissioningbrief/31971.
54. MacLeod C, Hudson J, Brogan M, et al. ScotCap - A large observational cohort study. Colorectal Disease 2022;24(4):411-21. doi: https://dx.doi.org/10.1111/codi.16029.
55. Möllers T, Schwab M, Gildein L, et al. Second-generation colon capsule endoscopy for detection of colorectal polyps: Systematic review and meta-analysis of clinical trials. Endosc Int Open 2021;9(4):E562-e71. doi:10.1055/a-1353-4849.
56. Kjølhede T, Ølholm AM, Kaalby L, et al. Diagnostic accuracy of capsule endoscopy compared with colonoscopy for polyp detection: systematic review and meta-analyses. Endoscopy 2021;53(7):713-21. doi: 10.1055/a-1249-3938.
57. Scottish Health Technologies Group (SHTG). Second-generation colon capsule endoscopy (CCE-2) for the detection of colorectal polyps: SHTG recommendation. 2020. https://shtg.scot/media/1888/second-generationcolon-capsule-endoscopy-cce-2-for-the-detection-of-colorectal-polyps-shtg-recommendation-01-20-gastro.pdf.
58. Nishikawa T, Nonogaki K and Nakamura M. Capsule endoscopy reading support centers: current status and challenges Gastroenterological Endoscopy; 2022 64(3), pp. 239-248.
59. Semenov S, Costigan C, Ismail M et al Low Colon Capsule Endoscopy (CCE) False Negative Rate for Polyps Excluding Reader Error. Diagnostics; 2023 Jan; 13(1): 56. doi:10.3390/diagnostics13010056.
60. Donner A, Rotondi MA. Sample size requirements for interval estimation of the kappa statistic for interobserver agreement studies with a binary outcome and multiple raters. Int J Biostat. 2010;6(1):Article 31. doi: 10.2202/1557-4679.1275.
61. Deding U, Boggild H, Baatrup G et al Socioeconomic differences in expected discomfort from colonoscopy and colon capsule endoscopy Preventive Medicine; Prev Med. 2023 Jun 24;173:107593. doi:10.1016/j.ypmed.2023.107593.
62. Ismail MS, Murphy G, Semenov S et al. Comparing CCE to colonoscopy; a symptomatic patient's perspective BMC Gastroenterology; 2022; 22: 31. doi: 10.1186/s12876-021-02081-0.

63. MacLeod C, Dobson-McKittrick A and Watson AJM. Is there an association between patient factors and a successful colon capsule endoscopy test? - A retrospective cohort study Colorectal Disease. Conference:ACPGBI Annual Meeting. 2022 24 (Supplement 2) (pp 17).
64. Chu H, Cole S. Sample size calculation using exact methods in diagnostic test studies. J Clin Epidemiol. 2007 Nov;60(11):1201-2. doi: 10.1016/j.jclinepi.2006.09.015.
65. Umemneku Chikere CM, Wilson K, Graziadio S et al. Diagnostic test evaluation methodology: A systematic review of methods employed to evaluate diagnostic tests in the absence of gold standard – An update. 2019 PLoS ONE 14(10): e0223832. https://doi.org/10.1371/journal.pone.0223832.
66. Smith CT, Hopkins C, Sydes M, et al. Good practice principles for sharing individual participant data from publicly funded clinical trials. Trials. 2015;16(S2):O1. doi:10.1186/1745-6215-16-S2-O1.

17 APPENDICIES

17.1 Principal Investigator responsibilities

The PI's legal responsibilities are clearly defined in the UK Policy Framework for Health and Social Care Research & Clinical Trials Regulations.

The PI takes responsibility for the conduct of the research at the site and must ensure that the research is conducted according to the approved protocol and in compliance with any applicable regulatory standards and guidance. This includes:

- Attending the site initiation visit
- Ensure new members of the trial team are appropriately trained in the protocol and its procedures
- Ensuring that the ISF is accurately maintained
- Disseminating important safety or trial related information to all stakeholders within their site
- Ensuring that safety reporting is completed within the timelines
- Ensuring data is submitted to the eCRF in a timely manner
