# Supplementary material for: ColoCap: determining the diagnostic accuracy of colon capsule endoscopy compared with standard colonoscopy in patients at risk of colorectal disease – a study protocol
Source: BMJ Open. 2025 Sep 30;15(9):e104661. doi: 10.1136/bmjopen-2025-104661 (PMC12506084; doi:10.1136/bmjopen-2025-104661)
Supplement: Supplementary file 2 [file bmjopen-2025-104661.reviewer_comments.pdf]

PEER REVIEW HISTORY

BMJ Open publishes all reviews undertaken for accepted manuscripts. Reviewers are asked to complete a checklist review form and are provided with free text boxes to elaborate on their assessment. These free text comments are reproduced below.

ARTICLE DETAILS

Title (Provisional)

ColoCap study protocol: determining the diagnostic accuracy of colon capsule endoscopy compared to standard colonoscopy in patients at risk of colorectal disease

Authors

Ibrahim, Hussain; Haritakis, Monica; Ballantine, Lisa; McCormack, Kirsty; Cotton, Seonaidh; Hudson, Jemma; Atkin, Karl; Rogers, Stephen; Nixon, Lisette Sheena; Verghese, Angel; Holmes, Hayden; Treweek, Shaun; MacLennan, Graeme; Dolwani, Sunil; Gardner, Georgina; Hurt, Chris; Watson, Angus; Turvill, James

---

VERSION 1 - REVIEW

**Reviewer**	**1**
**Name**	**Rosa, Bruno**
**Affiliation**	**Senhora da Oliveira Hospital Guimaraes, Gastroenterology**
**Date**	**25-Jun-2025**
**COI**	**None**

Original investigation on a relevant clinical topic. Three parallel workstreams ensure proper evaluation of different outcomes

Thorough and well designed protocol.

Limitations were correctly identified and addressed

No major flaws that would prevent a sound interpretation of data have been identified

---

**Reviewer**	**2**
**Name**	**Liao, Juan**
**Affiliation**	**Sichuan University, digestive department**
**Date**	**30-Jun-2025**
**COI**	**None**

In protocol paper entitled “ColoCap study protocol: determining the diagnostic accuracy of colon capsule endoscopy compared to standard colonoscopy in patients at risk of colorectal disease”, this study aim to determine the diagnostic accuracy and cost effectiveness of colon capsule endoscope (CCE) compared to colonoscopy in cohorts. The following questions are list in below:

1. The exclusion criteria suggest to add “Unable to follow or tolerate fasting, and other study procedures”; “Known allergy or other known contraindications or intolerance to ingredients used in this study procedures”; “History of or suspicion for: volvulus or intestinal obstruction; fistula or diverticulum; internal hernias or megacolon that the Investigator considers as an exclusion”.

2. Measures of diagnostic accuracy suggest to include the accuracy of colon polyps, the accuracy of colon adenoma; ease of swallowing the capsule, quality of bowel images; the bowel cleansing quality. Ability of the CCE to identify the presence of diverticula or vascular malformation et al.

Reviewer 3

Name Garfinkle, Richard

Affiliation Jewish General Hospital, Division of Colon and Rectal Surgery

Date 02-Jul-2025

COI None

The authors have prepared a study protocol aimed at evaluating the diagnostic accuracy of colonic capsule endoscopy (CCE) as a means of diagnostic evaluation of the colon for mucosal abnormalities (as compared to colonoscopy, the standard of care). The study protocol is well written and organized. The study is important and will be of great interest to many subspecialties within medicine and surgery. I have the following comments:

1) Inclusion criteria - this needs to be clarified. "Patients with suspected CRC who have had a faecal immunochemical test (FIT) within 3 months" - does this mean patients with a positive FIT from the average-risk screening population? Does it exclude patients with other CRC symptoms (I would imagine so, if they had a FIT, given FIT is not usually performed as a diagnostic test). Also, "new IBD colitis suspected" may overlap with the exclusion criterion of "symptomatic patients with suspected CRC who have not had a FIT

within 3 months of referral." Does a patient with rectal bleeding fit the inclusion criterion or the exclusion criterion?

2) Based on the literature, what was the reason for patients (10%) not wanting to proceed with a colonoscopy after the CCE?

3) How do the authors define a "completed and adequately prepared" CCE or colonoscopy? Is it based on the Boston bowel prep score? Does the same rule apply to CCE as it does for colonoscopy?

4) Among the 30 possibly participating centers, how many of them have an active CCE program or are able to start a CCE program soon? I worry about the planned recruitment rate.

5) For WS2 (intra- and inter-observe variability), how was the number 140 chosen for CCE's?

VERSION 1 - AUTHOR RESPONSE

Reviewer: 1

Dr. Bruno Rosa, Senhora da Oliveira Hospital Guimaraes

Comments to the Author:

Original investigation on a relevant clinical topic. Three parallel workstreams ensure proper evaluation of different outcomes

Thorough and well designed protocol.

Limitations were correctly identified and addressed

No major flaws that would prevent a sound interpretation of data have been identified.

Response:

We appreciate you taking the time to consider the manuscript. We hope the findings of this study will add important context for patients consenting to undergo CCE and for the units and health systems considering the introduction of, or currently offering, CCE.

Reviewer: 2

Dr. Juan Liao, Sichuan University

Comments to the Author:

In protocol paper entitled “ColoCap study protocol: determining the diagnostic accuracy of colon capsule endoscopy compared to standard colonoscopy in patients at risk of colorectal disease”, this study aim to determine the diagnostic accuracy and cost effectiveness of colon capsule endoscope (CCE) compared to colonoscopy in cohorts. The following questions are list in below:

1. The exclusion criteria suggest to add “Unable to follow or tolerate fasting, and other study procedures”; “Known allergy or other known contraindications or intolerance to ingredients used in this study procedures”; “History of or suspicion for: volvulus or intestinal obstruction; fistula or diverticulum; internal hernias or megacolon that the Investigator considers as an exclusion”.

2. Measures of diagnostic accuracy suggest to include the accuracy of colon polyps, the accuracy of colon adenoma; ease of swallowing the capsule, quality of bowel images; the bowel cleansing quality. Ability of the CCE to identify the presence of diverticula or vascular malformation et al.

Response:

Thank you for your comments, Dr Liao.

In point 1, you suggested adding the following to the exclusion criteria:

- Unable to follow or tolerate fasting, and other study procedures

- Known allergy or other known contraindications or intolerance to ingredients used in this study procedures

- History of or suspicion for: volvulus or intestinal obstruction; fistula or diverticulum; internal hernias or megacolon that the Investigator considers as an exclusion

The authors agree the above scenarios warrant exclusion from the study. It is felt the current criteria listed below sufficiently cover the above scenarios:

- “Patients who are unable to safely swallow the CCE” *, “Patients who are unable to safely and fully comply with the bowel preparation” *, “Patients who will not be able to safely tolerate the study” *.

The * leads the reader to the following paragraph where your comments have been addressed in a new underlined sentence:

“These exclusion criteria will require some clinical judgement in line with the existing approach to CCE and colonoscopy in clinical practice. Judgement of ability to tolerate the study requires an assessment of frailty per se, rather than a specific co-morbidity. However, it is likely to include patients with conditions such as cirrhosis, diabetes, stroke, peripheral

vascular, heart or renal disease or cognitive impairment(29). They are also likely to exclude those with allergies to the medication used in the preparation, those who will not be able to comply with study procedures, and those with structural or functional intestinal pathologies such as Volvulus, severe adhesions, enteric fistula, or megacolon, for example."

In point 2, you suggested the addition of the following as measures of diagnostic accuracy:

- colon polyps
- colon adenoma
- ease of swallowing the capsule
- quality of bowel images
- bowel cleansing quality
- Diverticula or vascular malformation

It is recognised that a proportion of patients will not tolerate swallowing the capsule and will have poor cleansing/poor image quality. The power calculation has taken this into account. We hope to identify the diagnostic accuracy of CCE in those who have a complete and adequately cleansed and imaged colon. Colonic polyps/adenomas are already included in the diagnostic accuracy assessment.

The presence of other colonic pathologies will be recorded for both CCE and colonoscopy but have not formed part of the power calculations...

Reviewer: 3

Dr. Richard Garfinkle, Jewish General Hospital

Comments to the Author:

The authors have prepared a study protocol aimed at evaluating the diagnostic accuracy of colonic capsule endoscopy (CCE) as a means of diagnostic evaluation of the colon for mucosal abnormalities (as compared to colonoscopy, the standard of care). The study protocol is well written and organized. The study is important and will be of great interest to many subspecialties within medicine and surgery. I have the following comments:

1) Inclusion criteria - this needs to be clarified. "Patients with suspected CRC who have had a faecal immunochemical test (FIT) within 3 months" - does this mean patients with a positive

FIT from the average-risk screening population? Does it exclude patients with other CRC symptoms (I would imagine so, if they had a FIT, given FIT is not usually performed as a diagnostic test). Also, "new IBD colitis suspected" may overlap with the exclusion criterion of "symptomatic patients with suspected CRC who have not had a FIT

within 3 months of referral." Does a patient with rectal bleeding fit the inclusion criterion or the exclusion criterion?

2) Based on the literature, what was the reason for patients (10%) not wanting to proceed with a colonoscopy after the CCE?

3) How do the authors define a "completed and adequately prepared" CCE or colonoscopy? Is it based on the Boston bowel prep score? Does the same rule apply to CCE as it does for colonoscopy?

4) Among the 30 possibly participating centers, how many of them have an active CCE program or are able to start a CCE program soon? I worry about the planned recruitment rate.

5) For WS2 (intra- and inter-observe variability), how was the number 140 chosen for CCE's?

Response:

Thank you for your comments, Dr Garfinkle.

Point 1:

Patients taking part in national bowel screening programmes will not be included. Patients who present urgently to their GP and who have a FIT test will be included in the study. This is current UK practice.

There is overlap with CRC and IBD symptoms. Patients with these overlapping symptoms will be referred under the “suspected CRC” and will require FIT. Examples of suspected IBD colitis will include colitis on imaging, or those reviewed in clinic with a positive faecal calprotectin and symptoms of colitis (abdominal pain and PR blood and mucus, for example). This will be based on clinical judgement and different units will have different pathways for identifying those with suspected IBD.

Point 2:

Thank you “We anticipate 10% of participants that agree to take part will subsequently decline one or other of CCE and colonoscopy, as seen in comparable European studies”.

From the referenced papers:

Pecere et al. “Of these 222 patients, 19 (8.6%) refused to swallow the capsule, and when the AdvanCE system was used to position it in the stomach, the CCE-2 examination was interrupted”

Kobaek-Larsen et al. “45 withdrew their consent before the examinations were scheduled. Seven individuals were excluded before the examinations as they did not adhere to the bowel preparation protocol (n = 6) or were not able to swallow the video capsule (n = 1). One individual was excluded because consent was withdrawn subsequent to the CCE examination.”

Point 3:

Thank you; the following sentences have been added to the methods section:

“Bowel cleansing will be assessed using CC-CLEAR and Boston bowel preparation scores respectively in the CCE and colonoscopy reports.

“Adequate CCE completion is defined as a test that visualises the entire length of the colon and the anal cushions, or is excreted within the capsule’s battery life.”

“Colonoscopy is considered complete when the entire length of the colon is intubated as confirmed by features visualised in the caecum (appendix orifice, tri-radiate fold and the ileo-caecal valve).”

Point 4:

Thank you, all centres invited to participate have experience in performing CCE. Recruitment to trials is always a challenge but the Trial Management Team are confident the trial will reach its target. Recruitment is closely observed at our monthly meetings and further centres may be added if needed to meet our trial totals.

Point 5:

Thank you, the following line is included in the supplementary data:

“A prevalence of any mucosal lesion is expected to be 40%. If the agreement kappa is 0.9, a 95% confidence can be expected (one sided) that it is >0.82 with a sample size of 140 (kappa Size R package based on Donner and Rotondi 2010).”
